# Generalizable machine learning models for rapid antimicrobial resistance prediction in unseen health care settings

**DOI:** 10.1093/gigascience/giaf156

**Published:** 2026-01-19

**Authors:** Diane Duroux, Paul P Meyer, Giovanni Visonà, Niko Beerenwinkel

**Affiliations:** ETH AI Center, ETH Zurich, Andreasstrasse 5, 8092, Zurich, Switzerland; Department of Biosystems Science and Engineering, ETH Zurich, Klingelbergstrasse 48, 4056, Basel, Switzerland; Department of Quantitative Biomedicine, University of Zurich, Schmelzbergstrasse 26, 8006, Zurich, Switzerland; SIB Swiss Institute of Bioinformatics, Quartier UNIL-Sorge, 1015, Lausanne, Switzerland; Department of Mechanical and Process Engineering, ETH Zurich, Tannenstrasse 3, 8092, Zurich, Switzerland; Department of Empirical Inference, Max Planck Institute for Intelligent Systems, Max-Planck-Ring 4, 72076, Tübingen, Germany; Department of Biosystems Science and Engineering, ETH Zurich, Klingelbergstrasse 48, 4056, Basel, Switzerland; SIB Swiss Institute of Bioinformatics, Quartier UNIL-Sorge, 1015, Lausanne, Switzerland

**Keywords:** antimicrobial resistance, generalizability, machine learning, representation learning

## Abstract

**Background:**

The deployment of machine learning in clinical settings is often hindered by the limited generalizability of the models. Models that perform well during development tend to underperform in new environments, limiting their clinical utility. This issue affects models designed for the rapid identification of antimicrobial resistance, which is essential to guide treatment decisions. Traditional susceptibility tests can take up to 3 days, whereas integrating matrix-assisted laser desorption/ionization time-of-flight (MALDI-TOF) mass spectrometry with machine learning has the potential to reduce this to 1 day. However, model performance declines drastically in hospitals or time frames outside the training data.

**Results:**

To improve robustness, we develop advanced feature representations using masked autoencoders (MAEs) for MALDI-TOF spectra and chemical language models and SELF-referencing embedded strings (SELFIES) for antimicrobials. Cross-validated on data from 4 medical institutions, our models demonstrate improved performance and stability. The MAE and SELFIES encodings increase the area under the precision–recall curve by 4% when evaluated on unseen time periods, while the MAE and Molformer language model encodings improve it by 10% when applied across different hospitals.

**Conclusions:**

These results underscore the value of combining deep learning with chemical and spectral information to build generalizable, high-impact clinical artificial intelligence.

## Introduction

Antimicrobial resistance (AMR) presents a severe threat to public health, with an estimated 1.27 million deaths attributed to bacterial AMR in 2019 [1]. Improved care of severe infections and expanded access to antibiotics could prevent up to 92 million cumulative deaths between 2025 and 2050 [2]. Early identification of AMR is critical to improve the effectiveness of antimicrobial prescriptions and treatment [3]. Recent studies demonstrate that machine learning (ML) models leveraging matrix-assisted laser desorption/ionization time-of-flight (MALDI-TOF) mass spectrometry data of pathogens hold promise by reducing the time to identify antimicrobial resistance from 3 days to 1 day compared to traditional methods [4–6]. However, while these models achieve high performance when trained and tested in similar conditions, such as the same hospital and time frame, the models’ accuracy drops when applied to new contexts with (shifted) data from different hospitals or time frames [4]. Such declines in model performance can be particularly hard for the user to detect. The models’ predictions can evoke high confidence despite being incorrect, when models are applied to data from an unseen distribution, potentially misleading users and resulting in poor decision-making. Data shift can also introduce biases into model predictions, leading to unfair or inequitable outcomes for certain groups.

Low generalizability across clinical settings, where model performance declines when applied to different hospitals or regions, can stem from factors such as variations in patient demographics, microbial populations, data collection practices, or local health care protocols. These contextual differences cause the data distribution in a new location to diverge from that of the training data (i.e., a data shift), resulting in lower model performance. For example, pathogens and resistance mechanisms prevalent in one hospital or region may differ from those in another, complicating accurate AMR prediction across settings. Similarly, low temporal generalizability, reflected as a decrease in model performance when applied to data collected in different years, can arise from evolving microbial populations, changes in resistance mechanisms, or updates in health care practices over time. For instance, pathogens and resistance patterns may change over time, posing challenges for models trained on past data to reliably predict AMR in future settings. These temporal data shifts result in decreased model accuracy when applied to more recent data, challenging the ability of models to reliably predict AMR in dynamic clinical environments.

In zero-shot contexts, where hospitals lack the necessary infrastructure or data to develop their own ML models, traditional approaches to handling data shifts, such as domain adaptation, fine-tuning, and incorporating a hospital-specific variable, cannot be employed. Instead, data augmentation, continuous monitoring, and representation learning can help mitigate the effects of a data shift. Data augmentation enhances generalizability by generating synthetic samples to increase training data diversity. However, designing realistic synthetic data that accurately reflect real-world variability is challenging. Continuous monitoring of model performance is also essential for early detection of a data shift. Regularly evaluating model accuracy allows for timely interventions to maintain performance.

Representation learning could offer a more effective and scalable solution for improving model performance in zero-shot scenarios. It mitigates the impact of limited or imbalanced data, common challenges in health care, by leveraging large datasets for pretraining. This process captures diverse patterns, reducing the risk of overfitting when applied to smaller datasets. Additionally, representation learning accommodates evolving data landscapes, as the extracted features should remain relevant even when protocols, equipment, or patient demographics change. In contrast, data augmentation often requires continuous adjustments to address such shifts. Finding learning representations that can be used in different domains is, however, not trivial. It is instead a crucial challenge to face for biomedical applications of machine learning. When data from the target domain are available, methods that rely on active corrections, such as transfer learning and domain adaptation [7, 8], offer widely adopted approaches to improve predictive performance. When no data from the target domain are given, it is still possible to attempt to learn representations that are robust to a domain change, which is the objective of domain generalization [9], a much more challenging and less-established methodology compared to the aforementioned approaches. Therefore, we investigate representation learning as a generalized and scalable approach to deal with data shifts. Representation learning is particularly well suited to health care settings with varying infrastructure, data quality, and privacy constraints.

Efforts to leverage ML for AMR predictions have explored different antimicrobial molecular representations [10, 11] (i.e., encoding molecules in machine-readable formats), including one-hot encoding, Morgan fingerprints, SMILES strings, DeepSMILES strings with 1-dimensional convolutional neural network (1D CNN), DeepSMILES strings with transformer, and DeepSMILES strings with a recurrent neural network [5, 6]. Experimental results indicate that performance differences associated with different antimicrobial encodings are generally minimal [6]. The authors suggest 2 potential reasons for this [6]: the training dataset may include too few antimicrobials to learn meaningful relations based on the components of the molecular structure of the antimicrobials, and very similar molecular structures can correspond to vastly different resistance profiles. Richer antimicrobial representations may hold potential to address these 2 limitations. For example, antimicrobial representations generated by unsupervised transformer-based language models pretrained on a large unlabeled corpus have enabled state-of-the-art results in many downstream, predictive tasks [12, 13]. These representations might include structural relations and highlight relevant differences, enabling models to leverage these broader molecular features for enhanced predictive accuracy. In addition, SELF-referencing embedded strings (SELFIES) [14] have facilitated and streamlined a wide range of applications in chemistry [15] but have not, to our knowledge, been applied in the context of MALDI-TOF–based AMR prediction.

Enriching the representation of pathogens is another promising way to enhance AMR prediction. MALDI-TOF mass spectrometry generates spectra by ionizing pathogen samples with a laser, accelerating the resulting ions through a time-of-flight analyzer, and measuring their mass-to-charge ratios. These spectra serve as unique molecular fingerprints of the pathogens. State-of-the-art AMR prediction models leverage raw MALDI-TOF spectra as representations of the pathogens and projected them to a lower-dimensional space. For example, Visonà et al. [5] have trained a classification multilayer perceptron (MLP) with residual skip-connections network for AMR prediction. The model first projects the spectra and antimicrobial features to the same dimension before concatenating the 2 vector representations and using them as the input of a final MLP classifier. De Waele et al. [6] have extracted spectra representations from a neural network for AMR prediction, with small- or medium-sized network variants typically performing best. Emerging techniques, such as masked autoencoders (MAEs) [16], allow one to enrich the representation of input data by training the MAEs to reconstruct missing information. Using a MAE could enable the model to construct richer spectra representations, which capture essential patterns and reduce noise.

This study aims to develop a novel model architecture to improve generalizability, reducing the need to develop separate models for each hospital or data collection year. We quantify the generalizability of AMR prediction models and examine the impact of different encodings on model performance in a zero-shot setting. Additionally, we assess how these encodings influence model stability when transitioning from a nonzero-shot to a zero-shot context. By addressing limitations in generalizability across clinical settings and years, our work aims to enhance predictive accuracy and contribute to more adaptable and reliable AMR diagnostics.

## Methods

### Problem definition and notation

**Figure 1:**
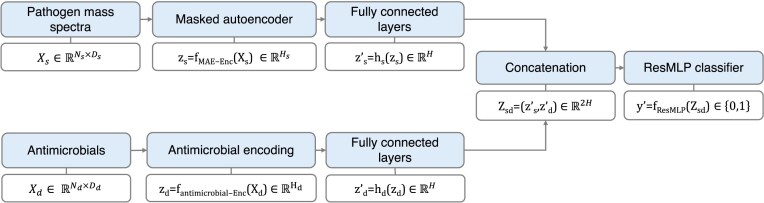
Model architecture: A masked autoencoder is applied to the binned representation of pathogen MALDI-TOF mass spectra ($D_s=6000$ and $H_s=512$). Antimicrobials are encoded using the Molformer transformer ($H_{\mathrm{d,Molformer}}=768$) or SELFIES ($H_{\mathrm{d,SELFIES}}=24160$). The pathogen and antimicrobial encodings are projected into a common latent space. These encodings are then concatenated to form a unified feature vector, which serves as input to a ResMLP classifier for final prediction.

We denote the MALDI-TOF mass spectra of pathogens by $\mathbf {X}_s \in \mathbb {R}^{N_s \times D_s}$, where $N_s$ is the number of pathogen spectra samples, and $D_s$ is the dimensionality of each spectrum. Antimicrobial drugs are represented by $\mathbf {X}_d \in \mathbb {R}^{N_d \times D_d}$, where $N_d$ is the number of drugs, and $D_d$ is the dimensionality of each antimicrobial molecular representation. For each pathogen–antimicrobial combination with an available susceptibility test outcome, a binary label $y \in \lbrace 0, 1\rbrace$ is provided, where 0 indicates sensitivity and 1 indicates resistance. To predict resistance versus susceptibility, we learn a function that maps each pair consisting of pathogen *i* and antimicrobial *j* to a binary label, $f(\mathbf {X}_s^i,\mathbf {X}_d^j) =: \hat{y}_{ij}$, approximating the true relationship. The primary objective of this study is to improve the representations of both input data types—mass spectra of pathogens and antimicrobial profiles—to improve the prediction of resistance or sensitivity *y* of each pathogen to a given antimicrobial (Fig. 1). In particular, we aim to make the model more adaptable to new contexts, such as different data collection years or hospitals.

### Data and preprocessing

The publicly available DRIAMS dataset [17] consists of MALDI-TOF mass spectrometry data collected from patients in the Swiss health care system. This dataset includes data from 4 distinct diagnostic laboratories, collected from 2015 to 2018: University Hospital Basel (designated as DRIAMS-A), Cantonal Hospital Basel-Land (DRIAMS-B), Cantonal Hospital Aarau (DRIAMS-C), and the Viollier laboratory service provider (DRIAMS-D). Data bias exists within DRIAMS-A due to 1 particular workstation (HospitalHygene), where samples were captured using a different medium and skewed heavily toward resistant samples. Following Weis et al. [4], we excluded data from this workstation to mitigate potential biases.

The filtered dataset encompasses 54,283 unique mass spectra associated with 631,167 antimicrobial resistance phenotypes. It covers 65 antimicrobials. Each entry provides a pathogen mass spectrum derived from a patient sample, with annotations indicating susceptibility or resistance to antimicrobials. We used the 6,000-dimensional binned mass spectra vector representation, consistent with the approach of Weis et al. [4].

The dataset has a noticeable imbalance in the number of samples according to the hospital of origin (Supplementary Table S1). Specifically, A2017 has a substantially higher number of samples (179,334) compared to all other entries. Hospitals B, C, and hospital A with data collected in 2015 have fewer samples (32,377, 47,586, and 11,610, respectively).

### Mass spectra encoding

We compare 2 pathogen representations: binned MALDI-TOF mass spectra and encodings generated by MAEs. The spectra were binned using a bin size of 3 *m/z* units, effectively reducing dimensionality while preserving spectral patterns. This bin size is sufficiently small to separate mass peaks, while still being large enough to maintain computational efficiency [4].

We used a MAE to generate novel encodings of mass spectra for downstream classification tasks. This unsupervised approach involves masking out random bins in each spectrum and training the model to reconstruct the missing information. For each training spectrum, multiple masked copies are generated. By creating varied versions of each spectrum, we multiply the training data and expose the model to a variety of masked versions of each training sample. This variety may help the autoencoder learn resilient patterns by repeatedly encountering and reconstructing diverse masked inputs.

Let $\mathbf {X}_s^{\mathrm{masked}}$ represent the spectra data after random masking is applied. The training dataset consists of these masked spectra paired with their corresponding original versions, serving as reconstruction targets. The feed-forward MAE comprises 2 components:

- The encoder, $f_{\mathrm{MAE-Enc}}: \mathbb {R}^{D_s} \rightarrow \mathbb {R}^{H_s}$, is a single dense layer with 512 neurons and ReLU activation. It maps the masked spectra into a low-dimensional latent space of dimension $H_s$, learning a compact representation $\mathbf {z}_s$.
- The decoder, $f_{\mathrm{MAE-Dec}}: \mathbb {R}^{H_s} \rightarrow \mathbb {R}^{D_s}$, reconstructs the original spectra from the encoded representation using a dense layer with sigmoid activation, training the model to predict missing values and capture comprehensive patterns.

After performing a parameter search (Supplementary Table S4 and Supplementary Fig. S7), the optimal parameters were selected as follows: the number of copies per pathogen sample was set to 10, the mask ratio ranged from 0.2 to 0.5, the encoding dimension was 512, the number of epochs was 100, the batch size was 50, and the learning rate was 0.001.

Throughout training, the model learns to reconstruct the original spectra from masked inputs, prompting the encoder to develop a robust and generalizable feature representation. Once training is complete, we use the encoder to extract the encoded representations by processing the original, unmasked spectra data. The spectrum representation $\mathbf {z}_s$ for a given pathogen is $\mathbf {z}_s = f_{\mathrm{MAE-Enc}}(\mathbf {X}_s)$. This final representation serves as the input for downstream tasks.

To disentangle the effects of general representation learning from the specific contribution of masking, we trained a standard (nonmasked) autoencoder on the spectra and evaluated its performance using the same experimental setup. The comparison of models with and without masking, highlighting the gain achieved through the masking strategy, is presented in the supplementary section, “Mass spectra encoding.” Alternative MAE encoding techniques (using 1D CNNs and transformers instead of feed-forward networks) were evaluated but not retained, as they showed lower performance (Supplementary Fig. S6).

### Antimicrobial encoding

We compare 3 types of antimicrobial representations: traditional molecular fingerprints, encodings generated by the Molformer transformer-based model for molecular representation learning [12], and SELFIES [14].

Molecular fingerprinting [18] is a widely used technique in chemical informatics and antimicrobial discovery, converting molecular structures into numerical features for use in machine learning models. Fingerprints represent a molecule as a binary vector, where each bit indicates the presence or absence of specific substructures or chemical patterns. This approach captures different aspects of a molecule’s structure, including topological, physiochemical, and other structural properties, making it highly effective in various applications [19–21]. In Visonà et al. [5], 3 standard fingerprinting techniques were evaluated: the molecular ACCess system (MACCS) keys (166 bits) [22], the PubChem fingerprints (PubChemFP) (881 bits) [23], and the 1,024-bit Morgan fingerprints (1,024 bits) [24]. No single fingerprint class demonstrated consistent superiority over the others. The authors opted to use Morgan fingerprints due to their widespread adoption in small-molecule screening and proven robust performance across various tasks. Following this approach, we also use 1,024-bit Morgan fingerprints.

To capture more complex features of antimicrobials, we leverage Molformer [12], a large-scale transformer model pretrained for molecular representation learning. Unlike traditional fingerprinting, Molformer encodings are learned representations that aim to capture semantic relationships within molecular structures beyond discrete substructures. Molformer applies self-attention mechanisms across molecular graphs, allowing it to model interactions and dependencies among atoms and bonds. This structure may help capture features of molecular conformation, functional groups, and other chemical characteristics, providing a more comprehensive molecular encoding. Specifically, we used the available model that is pretrained on 10% of the Zinc and PubChem database. Each antimicrobial is encoded as a vector of dimension 768.

We also evaluate SELFIES [14], a string-based representation for molecules. This approach addresses a critical limitation of the standard molecular representation, SMILES [25], which often produces strings that do not correspond to valid molecules. In contrast, SELFIES overcomes this issue by ensuring that every SELFIES string represents a valid molecule and that every molecule can be represented using SELFIES. Starting with the SMILES representation of the antimicrobials in the DRIAMS dataset, we convert the SMILES strings into SELFIES. To ensure uniform encoding dimensions, we transform the SELFIES strings into one-hot encodings by first constructing an alphabet from the SELFIES strings. Each SELFIES string is then converted into a padded one-hot encoding, which is subsequently flattened. This process results in each antimicrobial being represented as a vector of dimension 24,160. We define the antimicrobial encoder (Molformer or SELFIES) as $f_{\mathrm{antimicrobial-Enc}}: \mathbb {R}^{D_d} \rightarrow \mathbb {R}^{H_d}$, where $H_d$ is the hidden dimensionality of the antimicrobial representation. The antimicrobial representation $\mathbf {z}_d$ for a given antimicrobial is the image $\mathbf {z}_d = f_{\mathrm{antimicrobial-Enc}}(\mathbf {X}_d)$.

### Model architecture

Since $H_s$ and $H_d$ differ, we introduce 2 projection layers: a projection for the mass spectrum encoding, $h_s: \mathbb {R}^{H_s} \rightarrow \mathbb {R}^{H}$, and a projection for the antimicrobial encoding, $h_d: \mathbb {R}^{H_d} \rightarrow \mathbb {R}^{H}$. The projected encodings are then $\mathbf {z}_s^{\prime } = h_s(\mathbf {z}_s) \in \mathbb {R}^{H}$ and $\mathbf {z}_d^{\prime } = h_d(\mathbf {z}_d) \in \mathbb {R}^{H}$. After projecting both representations to the same dimensionality *H*, we concatenate $\mathbf {z}_s^{\prime }$ and $\mathbf {z}_d^{\prime }$ to form a combined representation $\mathbf {z}_{sd} = (\mathbf {z}_s^{\prime }, \, \mathbf {z}_d^{\prime }) \in \mathbb {R}^{2H}$.

In Visonà et al. [5], 3 primary models are explored: (i) encodings derived by applying principal component analysis to mass spectra and chemical fingerprints, which are concatenated and used as input for a logistic regression model; (ii) joint representations generated by Siamese networks, subsequently utilized as input for logistic regression to predict resistance; and (iii) a classification MLP with residual skip-connections. Building on the findings of Visonà et al. [5], which identified the MLP with residual skip-connections [26] as achieving the highest performance, we trained this network to predict the probability of resistance for antimicrobial–spectrum pairs. The concatenated vector $\mathbf {z}_{sd}$ is used as input to a ResMLP model to classify $\mathbf {z}_{sd}$ into resistant or sensitive categories. $f_{\mathrm{ResMLP}}: \mathbb {R}^{2H} \rightarrow \lbrace 0, 1\rbrace$ for classification $y^{\prime } = f_{\mathrm{ResMLP}}(\mathbf {z}_{sd})$. This model incorporates skip-connections, which provide a path for the gradients of the loss with respect to the model weight matrices to bypass certain layers during back-propagation and reach deeper layers in the network, typically enhancing the training process [27].

The final prediction pipeline is as follows (Fig. 1): (i) Encode the pathogen spectrum: $\mathbf {z}_s = f_{\mathrm{MAE-Enc}}(\mathbf {X}_s)$. (ii) Encode the antimicrobial: $\mathbf {z}_d = f_{\mathrm{antimicrobial-Enc}}(\mathbf {X}_d)$. (iii) Project both encodings and concatenate the projections: $\mathbf {z}_s^{\prime } = h_s(\mathbf {z}_s)$, $\mathbf {z}_d^{\prime } = h_d(\mathbf {z}_d)$, and $\mathbf {z}_{sd} = (\mathbf {z}_s^{\prime }, \, \mathbf {z}_d^{\prime })$. (iv) Classify with ResMLP: $y^{\prime } = f_{\mathrm{ResMLP}}(\mathbf {z}_{sd})$.

### Performance assessment

We compared the baseline antimicrobial resistance model, which uses as input a 6,000-dimensional binned MALDI-TOF mass spectra vector for pathogen representation and Morgan fingerprints for antimicrobial encoding, to the competitive approaches outlined in the Introduction. In total, we compare 6 input configurations, combining 3 antimicrobial representations (Morgan fingerprints, MoLFormer, and SELFIES) with 2 spectra encodings (binned MALDI-TOF and masked autoencoder-based representations).

We designed 2 data splits to reflect different data-generating processes and examine the prediction capabilities of the previously described machine learning models. Notably, to prevent data leakage, each data split was designed so that no pathogen appeared in both the training and test sets.

1. *Hospital zero-shot split:* Select data from the DRIAMS dataset collected in 2018. Let *i*, $j \in \lbrace A, B, C, D \rbrace$ represent the hospitals in the dataset. Train the model on data from hospital *i*. Test the model on data from hospital *j*, where $j \ne i$.
2. *Year zero-shot split:* Select data from the DRIAMS A. Let *i*, $j \in \lbrace 2015, 2016, 2017, 2018\rbrace$ represent the year of data collection. Train the model on data from year *i*. Test the model on data from year *j*, where $j \ne i$.

We report 3 standard classification metrics for imbalanced data: area under the precision–recall curve (AUPRC), balanced accuracy, and the Matthews correlation coefficient (MCC).

## Results

We illustrate the generalizability challenges of the baseline antimicrobial resistance model [5], which utilizes a 6,000-dimensional binned MALDI-TOF mass spectra vector representation for pathogens and Morgan fingerprints for antimicrobial representations. We then investigate how alternative representations of both MALDI-TOF spectra and antimicrobials affect zero-shot prediction performance and evaluate whether the most effective encodings lead to improved model stability when transitioning from nonzero-shot to zero-shot settings (Fig. 1).

### Baseline AMR model performance substantially declines in zero-shot scenarios

**Figure 2:**
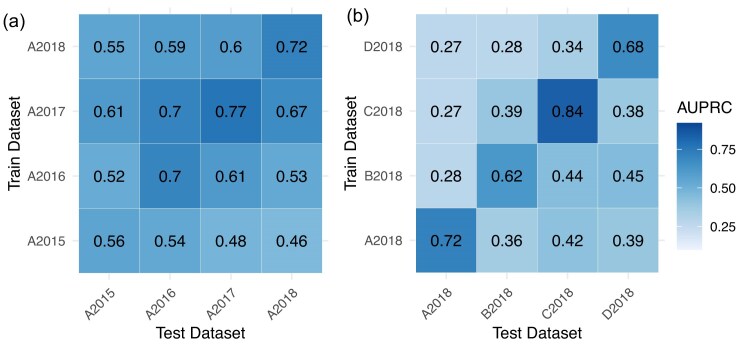
AUPRC across (a) years of data collection or (b) hospitals, with pathogens represented with 6,000-dimensional binned MALDI-TOF mass spectra vectors and antimicrobials represented with Morgan fingerprints. For reference, the resistant-to-susceptible class ratio is 0.19 for A2015, 0.23 for A2016, 0.19 for A2018, 0.17 for A2018 and B2018, 0.25 for C2018, and 0.15 for D2018.

The baseline model achieves the highest AUPRC values when trained and tested on data collected within the same year (Fig. 2a) for 3 of the 4 test sets, with AUPRC scores ranging from 0.56 for 2015 data to 0.77 for samples from 2017, indicating stable performance in a consistent temporal context. However, performance typically declines when the model is applied across different years, highlighting its sensitivity to temporal variations. On average, AUPRC decreases by 0.10 when tested on 2016 samples using models trained on data from other years. For 2017, it is 0.21; for 2018, the decrease is 0.16.

A similar pattern emerges with spatial generalizability, though the effect is even more pronounced. The model demonstrates robust performance when trained and tested on data from the same hospital, achieving AUPRC values ranging from 0.62 for hospital B to 0.84 for hospital C. However, performance drops when the model is trained on data from 1 hospital and tested on another, with AUPRC values consistently falling below 0.45. On average, the AUPRC decreases by 0.44 when tested on data from hospital A (2018) and trained on data from other years. For hospital B, the average decrease is 0.28; for hospital C, it is also 0.44; and for hospital D, the decrease is 0.27.

Overall, these findings highlight the challenges the AMR model faces in adapting across different hospitals and years. Additional plots for MCC and balanced accuracy are available in the supplementary materials.

### Advanced feature representation boosts model performance in year and hospital zero-shot contexts

**Figure 3:**
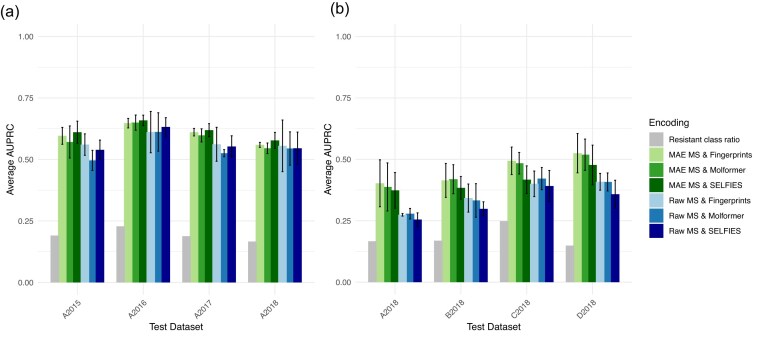
Average AUPRC across different test sets for (a) year-zero-shot (b) hospital-zero-shot analyses. Error bars represent standard deviation across training sets.

We analyze how various encoding methods impact model performance when no hospital-specific or year-specific data are available for training (zero-shot setting). We evaluate how well different encodings enable generalization to unseen hospitals or unseen years of data collection. We illustrate the generalizability of the model across clinical settings and years, measured by the average AUPRC across training sets in Fig. 3. The baseline corresponds to the light blue bars. Since the AUPRC of a random classifier is equal to the proportion of the positive class in the dataset, we indicate the proportion of resistant labels in each test set for reference. It ranges from 0.15 for D2018 to 0.25 for C2018.

Figure 3A presents the AUPRC of the model when trained on data from specific years and tested on datasets from different years. The test datasets correspond to hospital A, with samples collected in 2015, 2016, 2017, and 2018. Across all test cases, the baseline is consistently outperformed using alternative encodings, with the most substantial improvement attributed to the new pathogen representation. Replacing binned spectra with MAE encodings enhances AUPRC by 4% on average across the 4 test cases. MAE encodings also tend to reduce variability in model performance across different training sets, particularly for A2016, A2017, and A2018. Incorporating an advanced antimicrobial representation alongside MAE encodings further improves performance. Overall, using MAE encodings for pathogen representation and SELFIES representations for antimicrobial enhanced year-to-year generalizability. In our dataset, this approach led to an average performance increase of 4% (57.2 to 61.7) compared to the baseline.

Figure 3b presents the AUPRC of the model when tested on samples from a hospital not included in the training set. The hospital generalizability task is inherently more challenging than the temporal generalizability task, as evidenced by the decline in AUPRC between Figs. 3a and 3b. Despite this challenge, several alternative encoding techniques outperformed the baseline, primarily due to the MAE encodings of the pathogens. Replacing binned spectra with MAE encodings led to an average AUPRC increase of 9% across the 4 test cases. This improvement is more pronounced in the hospital generalization setting compared to the year-to-year generalization scenario. In the hospital generalization task, the most effective antimicrobial representations were Molformer encodings and Morgan fingerprints. Overall, using MAE encodings for pathogen representation combined with Molformer encodings or Morgan fingerprints for antimicrobial enhanced hospital-to-hospital generalizability, with an absolute increase in AUPRC of 10% (35.6 to 45.2) and 10% (35.6 to 45.9) respectively, compared to the baseline.

### Masked autoencoder pathogen encodings increase the nonzero-shot to zero-shot stability

**Figure 4:**
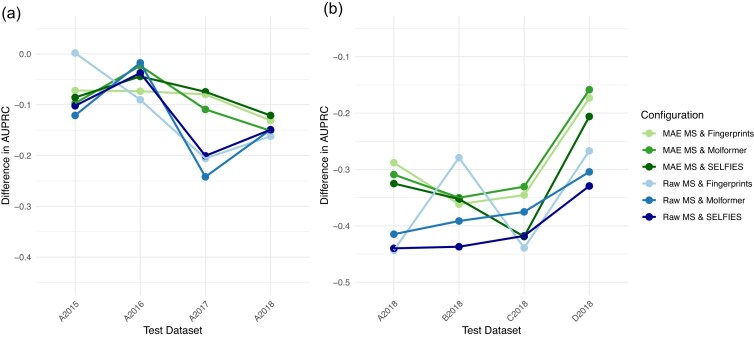
Zero-shot to nonzero-shot model stability: difference in AUPRC between performance obtained when the model is trained and tested on the same context, that is, (a) year and (b) hospital, versus trained and tested on difference contexts.

This section examines nonzero-shot to zero-shot (NS-ZS) stability (i.e., the differences in model performance when transitioning from a nonzero-shot to a zero-shot setting). We explore how encoding choices influence the gap between these 2 settings, highlighting which methods improve stability across different data availability scenarios. Assessing this NS-ZS stability will provide insights into the reliability of predictions in unseen contexts. Figure 4 illustrates the NS-ZS stability by showing a performance decrease when transitioning from a nonzero-shot context (model trained and tested on *i*) prediction scenario to a zero-shot scenario (model trained on *i* and tested on *j*, $j \ne i$). A smaller average decrease in AUPRC suggests a more stable model, indicating better generalizability across different contexts. The exact values and standard deviations obtained across training sets are available in Supplementary Tables S2 and S3.

The MAE encodings of the pathogen drive the highest improvement in model NS-ZS stability across time (Fig. 4a). On average, using MAE encoding increases the difference in AUPRC by 3% compared to raw binned spectra. When ranking the AUPRC difference for each test set and averaging these ranks, the top-performing encodings are MAE mass spectra combined with SELFIES antimicrobial or fingerprint encodings, with an average rank of 2.25. It is followed by MAE-Molformer, raw mass spectra–Molformer, raw mass spectra–SELFIES, and raw mass spectra–fingerprints. Thus, we identify MAE mass spectra combined with SELFIES or fingerprint antimicrobial encoding as the most stable configuration across temporal contexts. This approach reduces the NS-ZS mean AUPRC decrease from 11% with the baseline to 8% with MAE-Molformer and 9% with MAE-fingerprints, highlighting its effectiveness in enhancing generalizability.

Hospital NS-ZS stability remains a more complex challenge, as reflected in the larger absolute AUPRC difference between Fig. 4a (temporal NS-ZS stability) and Fig. 4b (hospital NS-ZS stability). In the hospital zero-shot scenario, we observe the same overall trend as in the temporal zero-shot scenario, where the main improvement in model NS-ZS stability is driven by MAE encodings of the pathogen—but to an even greater extent. On average, using MAE encoding increases the NS-ZS difference in AUPRC by 8% compared to raw binned spectra. When ranking the AUPRC difference across test sets and averaging these ranks, the top-performing encoding in this setting is MAE mass spectra combined with Molformer, followed by MAE-fingerprints, MAE-SELFIES, raw mass spectra–Molformer, raw mass spectra–fingerprints, and raw mass spectra–SELFIES. Thus, we identify MAE mass spectra combined with Molformer fingerprints as the most stable configuration across hospitals. This approach reduces the mean AUPRC decrease from 36% with the baseline to 29%.

### Maximizing generalizability by increasing variability and sample size in training sets

We compare the impact of training a model with data from 1 hospital versus 3 hospitals in the hospital zero-shot scenario. In the following, we focus on the combination of MAE and Molformer encodings, as well as MAE and Morgan fingerprint encodings, as these contribute the most to improving generalizability (Sections Advanced feature representation boosts model performance in year and hospital zero-shot contexts and Masked autoencoder pathogen encodings increase the non-zero-shot to zero-shot stability). Training on data from 3 hospitals not only increases the sample size but also introduces greater variability in the training set. In clinical decision support tools, aggregating data from multiple hospitals could be detrimental due to excessive data shifts. This issue arises in the AMR prediction task in the nonzero-shot (NS) scenario, where the test samples come from a hospital included in the training set. For instance, when using the MAE for pathogen encoding, training on a single hospital can be more beneficial than using data from 3 hospitals (Supplementary Fig. S5).

**Figure 5:**
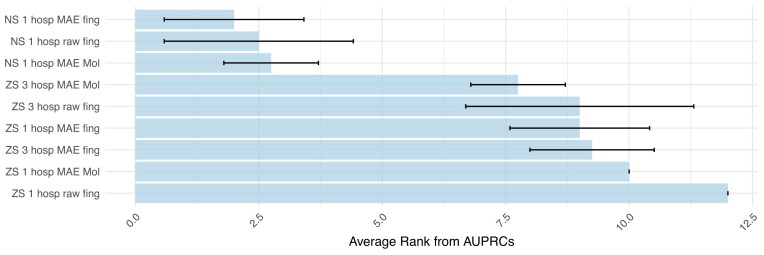
Average ranks across 4 test hospitals (A, B, C, and D) based on AUPRC. Nonzero shot (NS) means the test hospital was part of the training set, while hospitals in training and testing are different for the zero-shot (ZS) scenario. The confidence intervals show the standard deviation across the 4 test hospitals. Training was done using either 1 or 3 hospitals. When only 1 hospital was used in the ZS setting, AUPRC ranks were averaged over 3 models (e.g., for test hospital D, models trained on A, B, and C were averaged). Similarly, in the NS setting with 3 hospitals, ranks were averaged over 3 models trained on different hospital combinations (e.g., ABD, BCD, and ACD for test hospital D).

In the hospital zero-shot (ZS) scenario, this pattern does not hold. Instead, incorporating data from 3 hospitals consistently improves model performance compared to using data from 1 hospital (Fig. 5). On average, across 4 test hospitals, training with 3 hospitals enhances AUPRC performance by 11% when using baseline pathogen and antimicrobial encoding. Notably, this gain from increased data variability slightly exceeds the 10% improvement achieved by the best-performing encoding (MAE and Molformer). Additionally, combining data from multiple hospitals with robust encodings (MAE and Molformer) further enhances the model’s generalizability, yielding an average AUPRC increase of 12%.

When training the model with the baseline encodings on only 1 external hospital (ZS), the AUPRC decreased by 36% compared to training and testing on the same hospital data (NS). Incorporating data from 3 external hospitals along with robust encodings reduces the AUPRC decrease to 24%. In the ZS experiments, MAE and Molformer, using 3 external hospitals, achieved the highest score (Fig. 5). Overall, these findings highlight the importance of integrating robust encodings with data from multiple hospitals, whenever possible, to maximize model generalizability and performance in zero-shot contexts.

## Discussion

In this study, we aimed to enhance the generalizability of models to eliminate the need for developing a separate model for each hospital or each year of data collection. To overcome this challenge, we focus on creating robust input features that enhance the generalizability of models across different hospitals and time. In particular, we investigate the use of advanced representations for both pathogens and antimicrobials to improve the AMR model properties in zero-shot scenarios. Using masked autoencoder encodings for pathogens and SELFIES or transformer models for antimicrobials, we evaluate not only the overall improvement in predictive performance but also the enhanced stability of the models across different contexts. Our findings demonstrate that these alternative encodings outperform state-of-the-art methods for AMR prediction, offering a more robust and generalizable approach.

### Binned spectra

The binning of spectral data is a compromise between computational efficiency and expressive power. The specification of 6,000 bins corresponds to a bin size of  3 Da, which, as discussed in Weis et al. [4] (Methods—Spectral Representation), is small enough to still separate mass peaks. Furthermore, due to the methodology of data collection, the raw spectra may exhibit different cardinality, which would significantly complicate their use as inputs to the ML models. The binning approach is one of the simplest methods available to ensure uniform dimensionality for the samples. Also, the use of bins allows us to remove some contributions due to noisy measurements, which can introduce spurious signals in the data. Overall, the binning method used in the aforementioned study offered more advantages than disadvantages, and we opted to continue its use in our work.

**Strategies for antimicrobial encoding**. We utilized the pretrained Molformer transformer model to generate antimicrobial encodings due to its strong performance on classification and regression benchmarks from MoleculeNet [28]. We did not anticipate that fine-tuning Molformer would significantly improve results because the classifier had already encountered most antimicrobials or very similar ones during training. Furthermore, we used checkpoints from a Molformer model pretrained on a dataset of approximately 100 million molecules. This dataset consisted of 10% of molecules from ZINC and 10% from PubChem, originally used for training the Molformer-XL model. Access to a Molformer model trained on the full 100% dataset, rather than just a 10% subset, could potentially lead to further improvements in performance by providing more comprehensive encodings.

### Incorporating hospital-specific context

To improve the applicability of models across different hospitals, an additional step would be to identify factors that contribute to performance degradation across hospitals. Examples of such factors could include the type of MALDI-TOF machine used, the sample material (e.g., blood, urine, or tissue), the type of agar plate used for culturing before MALDI-TOF mass spectrometry analysis, or the preprocessing techniques employed in different laboratories. By including these contextual variables as additional features in the model, we could mitigate the variability in methodologies and infrastructure, thus improving generalizability. Training on a larger and more diverse dataset would improve the model’s robustness and enhance its ability to generalize across different environments. Unfortunately, these types of metadata are currently not available in publicly accessible datasets.

### Alternative strategies to handle data shift

In this work, we address the challenge faced by hospitals that lack the necessary infrastructure or data to develop their own machine learning models. Nevertheless, if hospitals had access to sufficient data and computational resources, alternative strategies could be employed to mitigate the effects of a data shift, including domain adaptation, fine-tuning, and incorporating hospital-specific variables. Domain adaptation techniques aim to align the distributions of source and target domains by minimizing discrepancies between them. This can be achieved through methods such as adversarial training, where a model learns to make predictions while an adversarial component ensures that source and target features become indistinguishable, or importance weighting, which adjusts the influence of source samples based on their relevance to the target domain. Fine-tuning involves training a model on data from 1 hospital and refining it with a small set of observations from a new hospital. This helps adapt the model to local variations. Another strategy is to incorporate a variable representing the hospital itself into the model, allowing it to account for institution-specific differences. While these approaches can improve model performance, they come with logistical challenges, such as requiring access to target domain data, which is often restricted in health care due to privacy constraints. Still, if access to new hospital data were available, these methods could be combined with representation learning to further enhance performance.

### Limitations of the study

The approach presented has several additional limitations. First, the use of alternative encodings, such as MAE, Molformer, or SELFIES, reduces the interpretability of the results. For example, when using MALDI-TOF mass spectrometry data, it is possible to identify specific features (*m/z* units) that contribute most to antimicrobial resistance prediction. This, in turn, can provide insights into which genes may be involved in resistance mechanisms. Similarly, Morgan fingerprints can be traced back to specific chemical substructures, allowing for a more interpretable link between molecular structure and model predictions. These interpretability advantages are diminished when using the more abstract encodings. To enhance interpretability, we recommend the combination of several *post hoc* methodologies that can be applied to deep learning models, as no single method may be reliable enough to provide robust conclusions. One of the simplest of such methods would be a *ceteris paribus* comparison, where each feature is varied while keeping the rest fixed [29]. More nuanced approaches include, for example, Integrated Gradients [30], DeepLift [31], and SHAP DeepExplainer [32].

Second, the use of MALDI-TOF mass spectrometry inherently limits resistance detection to mechanisms that manifest as changes in the spectral profile. For example, resistance mechanisms involving protein expression changes may be detectable, while those relying solely on point mutations might go unnoticed. Moreover, while we evaluated our approach on data from 4 different hospitals, these institutions are geographically close (northern Switzerland). As a result, the models may still benefit from regional similarities. Applying the models to hospitals in more distant or diverse geographic locations could reveal additional generalizability challenges.

### Model use case

In many countries, the standard workflow for patients with suspected infections involves empiric antibiotic therapy in parallel to microbiological diagnostics. Samples are cultured, and within 24–48 hours, the infecting organism is identified by MALDI-TOF. An additional 24–48 hours are then required for antimicrobial susceptibility testing (AST), after which therapy is adjusted. The proposed ML tool aims to accelerate this process by providing a predicted AST profile at the time of species identification (24–48 hours). The clinician’s workflow remains unchanged, but alongside species identification, the laboratory would deliver an ML-based resistance prediction. This shortens the diagnostic process by 24–48 hours and supports earlier optimization of therapy, which is critical for therapeutic success. In the long term, such ML systems could provide ranked susceptibility predictions, integrating with clinical and epidemiological data to guide treatment. Two main use cases are envisioned: (i) improving initial treatment success by identifying the most likely effective antibiotics and (ii) enabling de-escalation to narrower-spectrum agents, thereby supporting antimicrobial stewardship.

### Clinical deployment

While the antimicrobial resistance model demonstrates promising performance across multiple hospitals, it is not yet ready for direct clinical deployment. Beyond legal and regulatory considerations, several key steps remain before it can be integrated into clinical practice. First, model performance could likely be improved by incorporating additional patient-level features that are routinely available to clinicians, such as prior antibiotic use, recent hospitalization, immunosuppression, or travel history. These factors may influence the risk of antimicrobial resistance and could enhance both the accuracy and clinical relevance of the model. Second, it is important to evaluate the model’s role within the broader context of antimicrobial stewardship. In particular, we should assess whether the model could support clinical decisions such as the de-escalation of broad-spectrum antibiotics in favor of narrower, more targeted therapies. Third, prospective validation in real-world clinical settings is necessary to assess the model’s robustness and generalizability beyond retrospective data. Addressing these aspects will be essential for transitioning from a proof-of-concept model to one that is clinically trustworthy and actionable.

## Availability of Source Code and R

Project name: ZeroShotAMR

- Project homepage: https://github.com/DianeDuroux/ZeroShotAMR
- Operating system(s): Platform independent
- Programming language: python
- Software: RRID:SCR_027224
- Computational workflow: 10.48546/workflowhub.workflow.1999.1
- Other requirements: pytorch_lightning, scikit_learn, shap, tensorflow, torch, tqdm
- License: GNU GPL v3.0

## Additional Files

**Supplementary Table S1**. Labels distribution per hospital and year.

**Supplementary Table S2**. Zero-shot to nonzero-shot model stability: difference in AUPRC (and standard deviation across train sets) between performance obtained when the model is trained and tested on the same year versus trained and tested on different years. *MAE MS* aggregates *MAE MS & Fingerprints, MAE MS & Molformer*, and *MAE MS & SELFIES*, while *Raw MS* aggregates *Raw MS & Fingerprints, Raw MS & Molformer*, and *Raw MS & SELFIES*.

**Supplementary Table S3**. Zero-shot to nonzero-shot model stability: difference in AUPRC (and standard deviation across train sets) between performance obtained when the model is trained and tested on the same hospital versus trained and tested on different hospitals. *MAE MS* aggregates *MAE MS & Fingerprints, MAE MS & Molformer*, and *MAE MS & SELFIES*, while *Raw MS* aggregates *Raw MS & Fingerprints, Raw MS & Molformer*, and *Raw MS & SELFIES*.

**Supplementary Table S4**. MAE performance metrics for different parameter configurations.

**Supplementary Fig. S1**. Matthew correlation coefficients across (1) years of data collection or (2) hospitals, with pathogens represented with 6,000-dimensional binned MALDI-TOF mass spectra vector representation and antimicrobials represented with Morgan fingerprints.

**Supplementary Fig. S2**. Balanced accuracy across (1) years of data collection or (2) hospitals, with pathogens represented with 6,000-dimensional binned MALDI-TOF mass spectra vector representation and antimicrobials represented with Morgan fingerprints.

**Supplementary Fig. S3**. Average MCC across different test sets for (a) year-zero-shot (b) hospital-zero-shot analyses. Error bars represent standard deviation across training sets.

**Supplementary Fig. S4**. Average balanced accuracy across different test sets for (a) year-zero-shot (b) hospital-zero-shot analyses. Error bars represent standard deviation across training sets.

**Supplementary Fig. S5**. Average ranks across 4 test hospitals (A, B, C, and D) based on AUPRC, MCC, and balanced accuracy. Nonzero (NS) shot means the test hospital was part of the training set, while hospitals in training and testing sets are different in the zero-shot (ZS) scenario. The confidence intervals show the standard deviation across the 4 test hospitals. Training was done using either 1 or 3 hospitals. When only 1 hospital was used in the ZS setting, AUPRC ranks were averaged over 3 models (e.g., for test hospital D, models trained on A, B, and C were averaged). Similarly, in the NS setting with 3 hospitals, ranks were averaged over 3 models trained on different hospital combinations (e.g., ABD, BCD, and ACD for test hospital D).

**Supplementary Fig. S6**. Average AUPRC across test sets A2018, C2018, and D2018 for hospital-zero-shot analyses (training performed on B2018) for different encoding architectures.

**Supplementary Fig. S7**. Average AUPRC across test sets A2018, C2018, and D2018 for hospital-zero-shot analyses (training performed on B2018) for different embedding sizes.

**Supplementary Fig. S8**. Average AUPRC across test sets A2018, C2018, and D2018 for hospital-zero-shot analyses (training performed on B2018) for different embedding sizes.

## Abbreviations

1D CNN: 1-dimensional convolutional neural network; AMR: antimicrobial resistance; AST: antimicrobial susceptibility testing; AUPRC: precision–recall curve; MACCS: molecular ACCess system; MAE: masked autoencoder; MALDI-TOF: matrix-assisted laser desorption/ionization time-of-flight; MCC: Matthews correlation coefficient; ML: machine learning; MLP: multilayer perceptron; NS: nonzero shot; SELFIES: SELF-referencing embedded strings; ZS: zero shot.

## Supplementary Material

giaf156_Supplemental_File

giaf156_Authors_Response_To_Reviewer_Comments_original_submission

giaf156_GIGA-D-25-00242_original_submission

giaf156_GIGA-D-25-00242_Revision_1

giaf156_Reviewer_1_Report_original_submission César de la Fuente-Núñez -- 8/14/2025

giaf156_Reviewer_2_Report_original_submission Johan Lassen, Ph.D. -- 8/18/2025

giaf156_Reviewer_2_Report_revision_1 Johan Lassen, Ph.D. -- 11/10/2025

## Data Availability

The MALDI-TOF spectra used in this project can be obtained by downloading the DRIAMS dataset [17]. The code, data, and results presented in this article are available in the *GigaScience* repository, GigaDB [33]. The repository includes metadata, binned and MAE-preprocessed spectra, and drug encodings required to reproduce the analyses, along with a ZIP archive of the associated GitHub repository, the trained model weight files, and all data necessary to regenerate the figures.
