# Supplementary material for: Generalizable machine learning models for rapid antimicrobial resistance prediction in unseen health care settings
Source: Gigascience. 2026 Jan 19;15:giaf156. doi: 10.1093/gigascience/giaf156 (PMC12908719; doi:10.1093/gigascience/giaf156)
Supplement: giaf156_Supplemental_File [file giaf156_supplemental_file.pdf]

Generalizable machine learning models for rapid antimicrobial resistance prediction in unseen healthcare settings
Supplementary material

Table 1: Labels distribution per hospital and year

Dataset-Year	Antimicrobial resistance phenotypes
A-2015	11,610
A-2016	138,132
A-2017	179,334
A-2018	123,558
B-2018	32,377
C-2018	47,586
D-2018	98,570

Encoding	A2015	A2016	A2017	A2018
MAE MS & Fingerprints	-0.07 (0.03)	-0.07 (0.02)	-0.08 (0.02)	-0.13 (0.01)
MAE MS & Molformer	-0.10 (0.07)	-0.02 (0.03)	-0.11 (0.03)	-0.15 (0.02)
MAE MS & SELFIES	-0.09 (0.04)	-0.04 (0.02)	-0.07 (0.03)	-0.12 (0.03)
Raw MS & Fingerprints	0.00 (0.04)	-0.09 (0.08)	-0.21 (0.07)	-0.16 (0.10)
Raw MS & Molformer	-0.12 (0.04)	-0.02 (0.08)	-0.24 (0.01)	-0.15 (0.07)
Raw MS & SELFIES	-0.10 (0.04)	-0.04 (0.04)	-0.20 (0.04)	-0.15 (0.07)
**MAE MS**	-0.08 (0.01)	-0.05 (0.03)	-0.09 (0.02)	-0.13 (0.02)
**Raw MS**	-0.07 (0.07)	-0.05 (0.04)	-0.22 (0.02)	-0.15 (0.01)

Table 2: Zero-shot to non-zero-shot model stability: difference in AUPRC (and standard deviation across train sets) between performance obtained when the model is trained and tested on the same year, versus trained and tested on different years. *MAE MS* aggregates *MAE MS & Fingerprints*, *MAE MS & Molformer*, and *MAE MS & SELFIES*, while *Raw MS* aggregates *Raw MS & Fingerprints*, *Raw MS & Molformer*, and *Raw MS & SELFIES*.

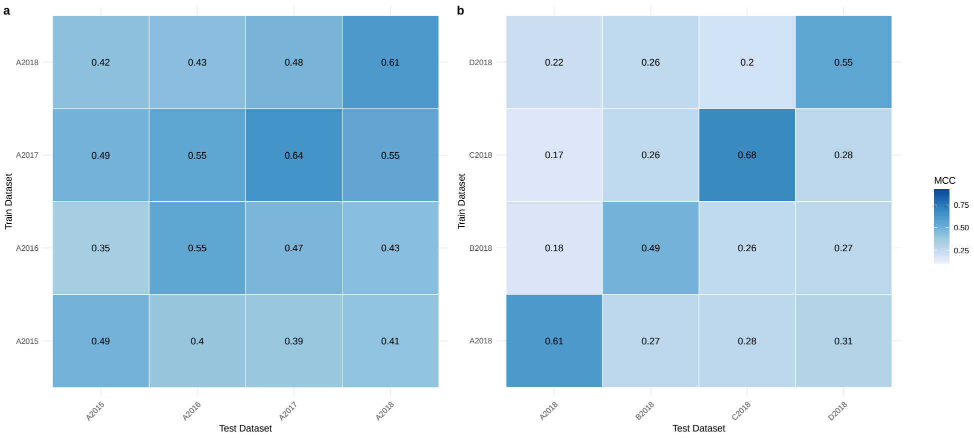

Fig. 1: Matthew correlation coefficients across (1) years of data collection or (2) hospitals, with pathogens represented with 6000-dimensional binned MALDI TOF mass spectra vector representation and antimicrobials represented with morgan fingerprints.

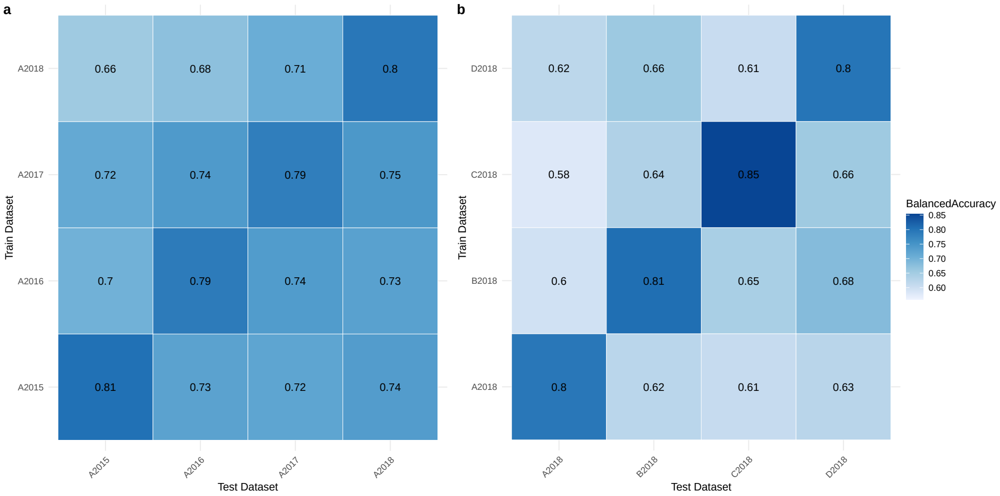

Fig. 2: Balanced accuracy across (1) years of data collection or (2) hospitals, with pathogens represented with 6000-dimensional binned MALDI TOF mass spectra vector representation and antimicrobials represented with morgan fingerprints.

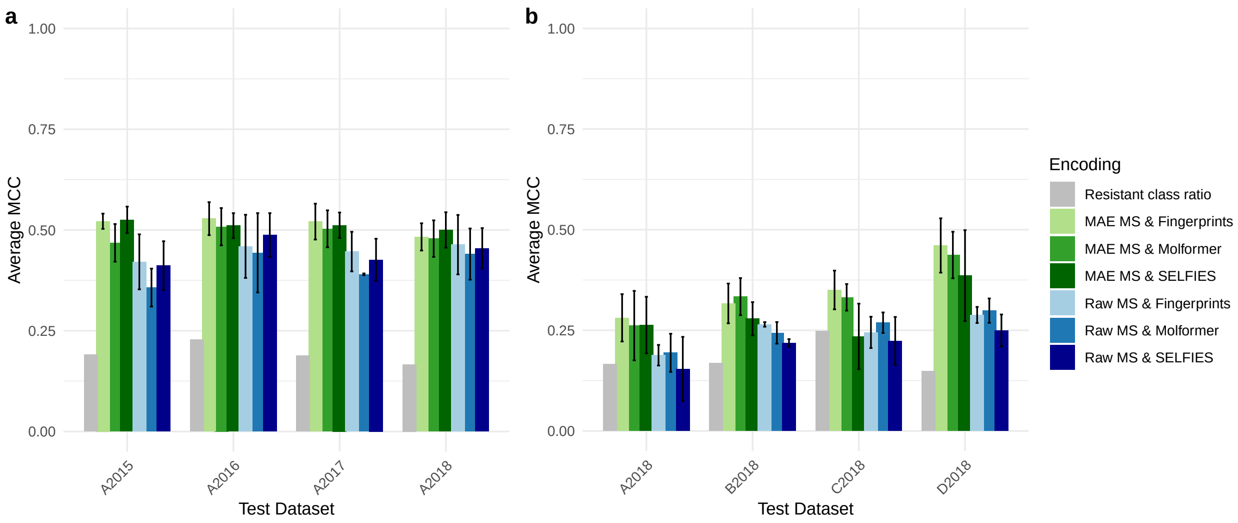

Fig. 3: Average MCC across different test sets for (a) year-zero-shot (b) hospital-zero-shot analyses. Error bars represent standard deviation across training sets.

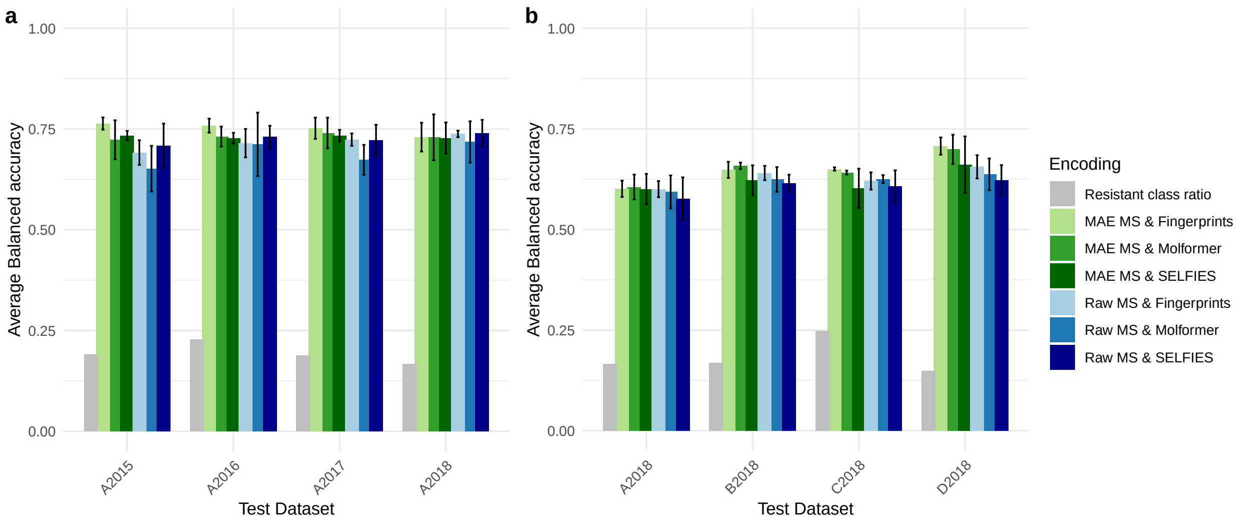

Fig. 4: Average balanced accuracy across different test sets for (a) year-zero-shot (b) hospital-zero-shot analyses. Error bars represent standard deviation across training sets.

**Encoding**	**A2018**	**B2018**	**C2018**	**D2018**
MAE MS & Fingerprints	-0.29 (0.10)	-0.36 (0.07)	-0.35 (0.06)	-0.17 (0.08)
MAE MS & Molformer	-0.31 (0.10)	-0.35 (0.06)	-0.33 (0.04)	-0.16 (0.06)
MAE MS & SELFIES	-0.32 (0.07)	-0.35 (0.05)	-0.42 (0.06)	-0.21 (0.08)
Raw MS & Fingerprints	-0.44 (0.01)	-0.28 (0.06)	-0.44 (0.05)	-0.27 (0.03)
Raw MS & Molformer	-0.41 (0.02)	-0.39 (0.07)	-0.38 (0.05)	-0.30 (0.04)
Raw MS & SELFIES	-0.44 (0.03)	-0.44 (0.03)	-0.42 (0.06)	-0.33 (0.06)
**MAE MS**	-0.31 (0.02)	-0.35 (0.01)	-0.36 (0.05)	-0.18 (0.02)
**Raw MS**	-0.43 (0.02)	-0.37 (0.08)	-0.41 (0.03)	-0.30 (0.03)

Table 3: Zero-shot to non-zero-shot model stability: difference in AUPRC (and standard deviation across train sets) between performance obtained when the model is trained and tested on the same hospital, versus trained and tested on different hospitals. *MAE MS* aggregates *MAE MS & Fingerprints*, *MAE MS & Molformer* and *MAE MS & SELFIES*, while *Raw MS* aggregates *Raw MS & Fingerprints*, *Raw MS & Molformer*, and *Raw MS & SELFIES*.

Table 4: MAE performance metrics for different parameter configurations

MR	Copy	Batch	Epoch	AUPRC	Balanced accuracy	MCC
0.01-0.2	10	50	100	76.5	79.8	63.7
0.5-0.7	10	50	100	76.7	78.7	62.5
0.01-0.2	100	50	10	77.0	81.4	61.8
0.2-0.5	100	50	10	77.3	79.8	64.8
0.2-0.5	100	50	100	76.2	80.6	65.1
0.5-0.7	100	50	100	77.7	80.2	64.8
0.2-0.5	2	50	100	78.1	78.9	65.1
**0.2-0.5**	**10**	**50**	**100**	**78.5**	**82.1**	**65.3**
0.1-0.7	10	50	100	75.6	80.9	61.9
0.4-0.6	10	50	100	78.6	80.7	64.0
0.2-0.5	20	50	100	73.8	79.1	60.6
0.2-0.5	10	500	100	77.9	81.8	63.8
0.2-0.5	10	5	100	77.9	91.8	66.0

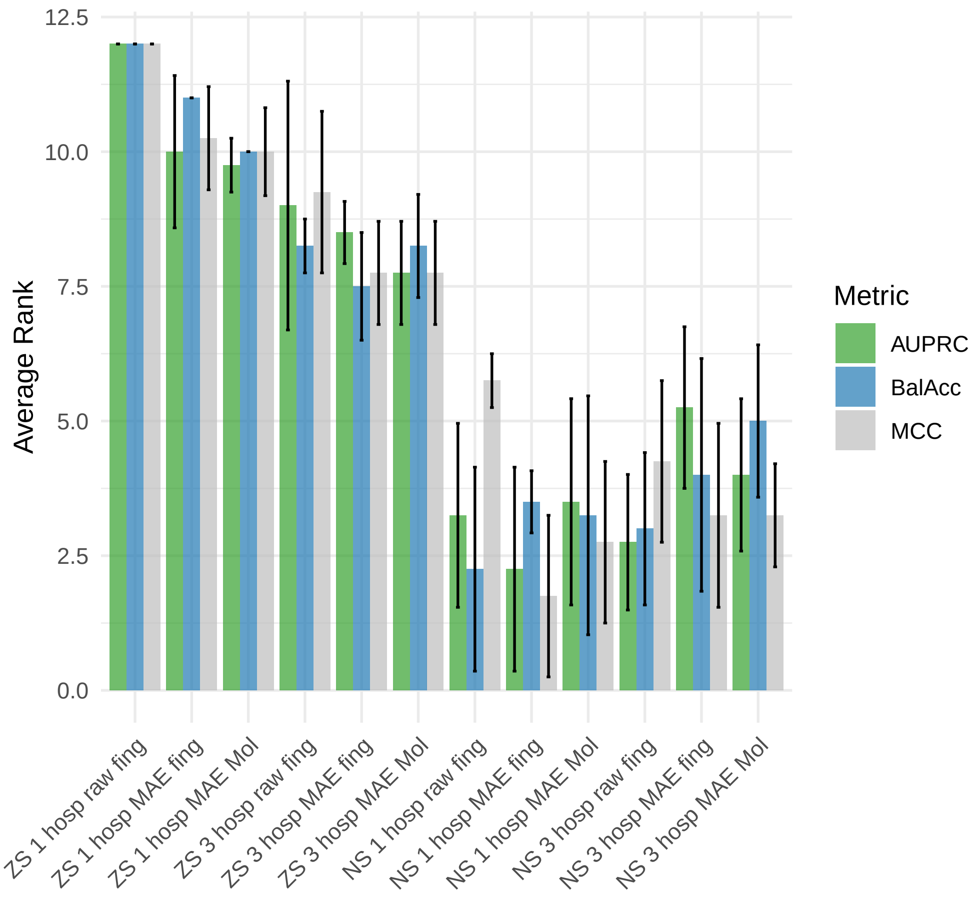

Fig. 5: Average ranks across four test hospitals (A, B, C, and D) based on AUPRC, MCC and balanced accuracy. Non-zero (NS) shot means the test hospital was part of the training set, while hospitals in training and testing sets are different in the zero-shot (ZS) scenario. The confidence intervals show the standard deviation across the four test hospitals. Training was done using either one or three hospitals. When only one hospital was used in the ZS setting, AUPRC ranks were averaged over three models (e.g., for test hospital D, models trained on A, B, and C were averaged). Similarly, in the NS setting with three hospitals, ranks were averaged over three models trained on different hospital combinations (e.g., ABD, BCD, and ACD for test hospital D).

Mass spectra encoding

To disentangle the effects of general representation learning from the specific contribution of masking, we trained a standard (non-masked) autoencoder on the same spectra and evaluated its performance using the same experimental setup 6. The comparison confirms that while the non-masked autoencoder does yield improvements over raw binned spectra, the masked autoencoder consistently outperforms the standard autoencoder across hospital test sets. This indicates that the gains observed in our study are not solely due to generic representation learning, but are indeed attributable to the masking strategy.

Also, the MAE architecture used to generate mass spectra embedding is a simple feedforward network with a single hidden layer. Given the structured nature of spectral data, more expressive architectures (e.g., 1D CNNs or transformers) could better capture local and contextual features. However, we identified that the performance of a 1D CNN or a transformer model was generally comparable to, and in most cases lower than that of our proposed MAE with a simple feedforward encoder (Fig. 6). The feedforward MAE consistently demonstrated robust and competitive performance across different hospital test sets. Overall, our benchmarking indicates that the simple feedforward MAE achieves performance on par with, and often exceeding, that of more complex architectures, suggesting that its expressive capacity is sufficient for the encoding task in this setting. These additional results have been included in the supplementary materials.

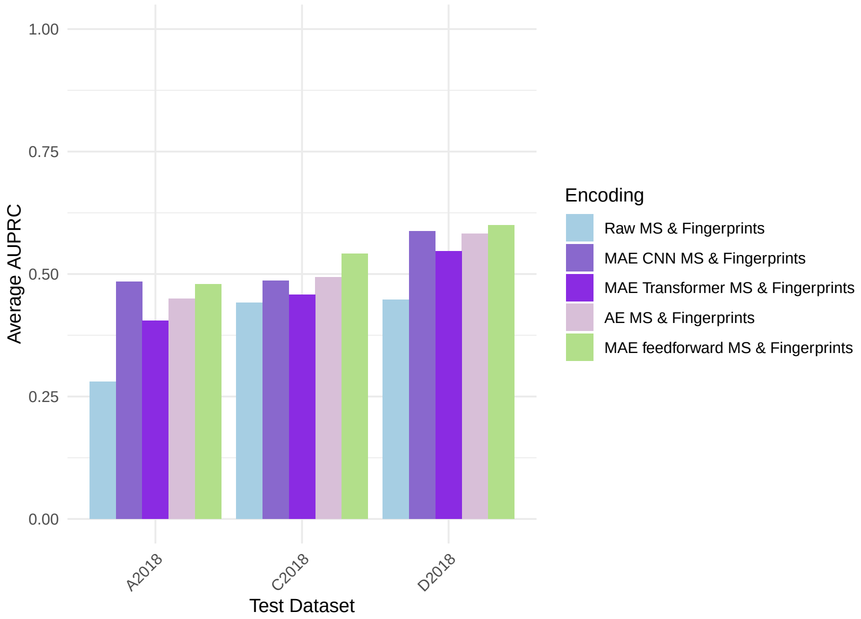

Fig. 6: Average AUPRC across test sets A2018, C2018 and D2018, for hospital-zero-shot analyses (training performed on B2018) for different encoding architectures.

We performed a sensitivity analysis to compare embedding sizes ranging from 128 to 2048 in a hospital zero-shot setting (trained on B2018 and tested on A2018, C2018, or D2018). Performance improves with increasing dimensionality up to 512, after which it plateaus and in some cases slightly declines (Fig. 7). This suggests that higher-dimensional embeddings do not provide additional benefit and may even introduce overfitting or noise. Based on these results, we selected 512 dimensions.

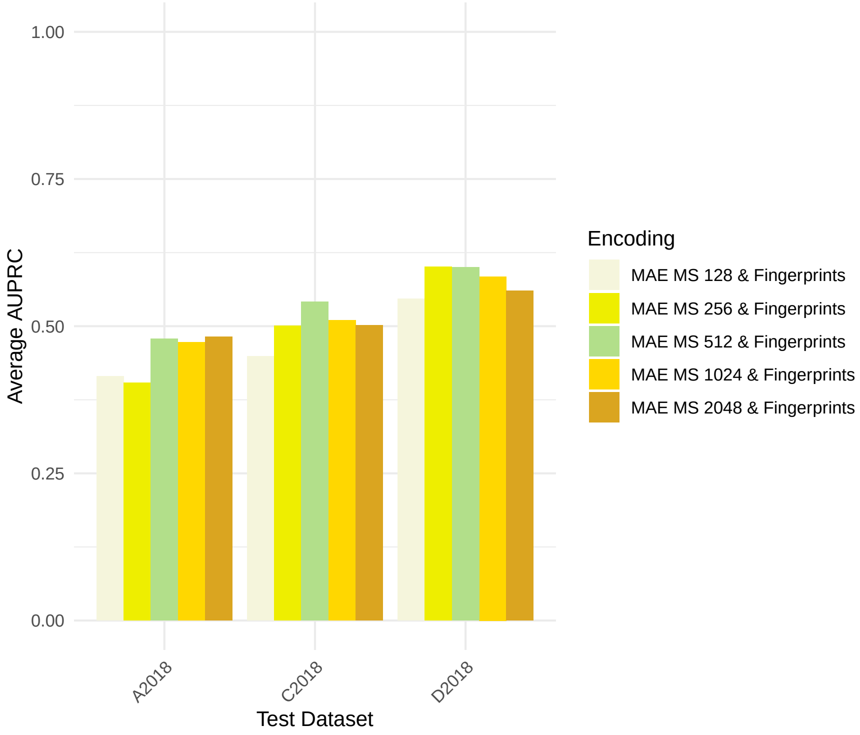

Fig. 7: Average AUPRC across test sets A2018, C2018 and D2018, for hospital-zero-shot analyses (training performed on B2018) for different embedding sizes.

Qualitative Analysis of Embeddings

We performed a UMAP analysis to assess technical bias with and without MAE embeddings. Using binned spectra (without MAE), samples from different datasets (i.e., hospitals) overlap extensively. While there are small local enrichments, overall batch effects are relatively minor, and spectra from different hospitals occupy similar regions of the feature space. In contrast, species-level structure is much more pronounced: distinct clusters emerge that correspond to different genera (e.g., *Escherichia*, *Klebsiella*, *Staphylococcus*, *Proteus*). This shows that binned spectra retain strong taxonomic signals, with spectra from the same genus grouping closely together. UMAP visualizations based on MAE embeddings show very similar patterns, suggesting that MAE also minimizes technical variation while robustly capturing biological, genus-level distinctions.

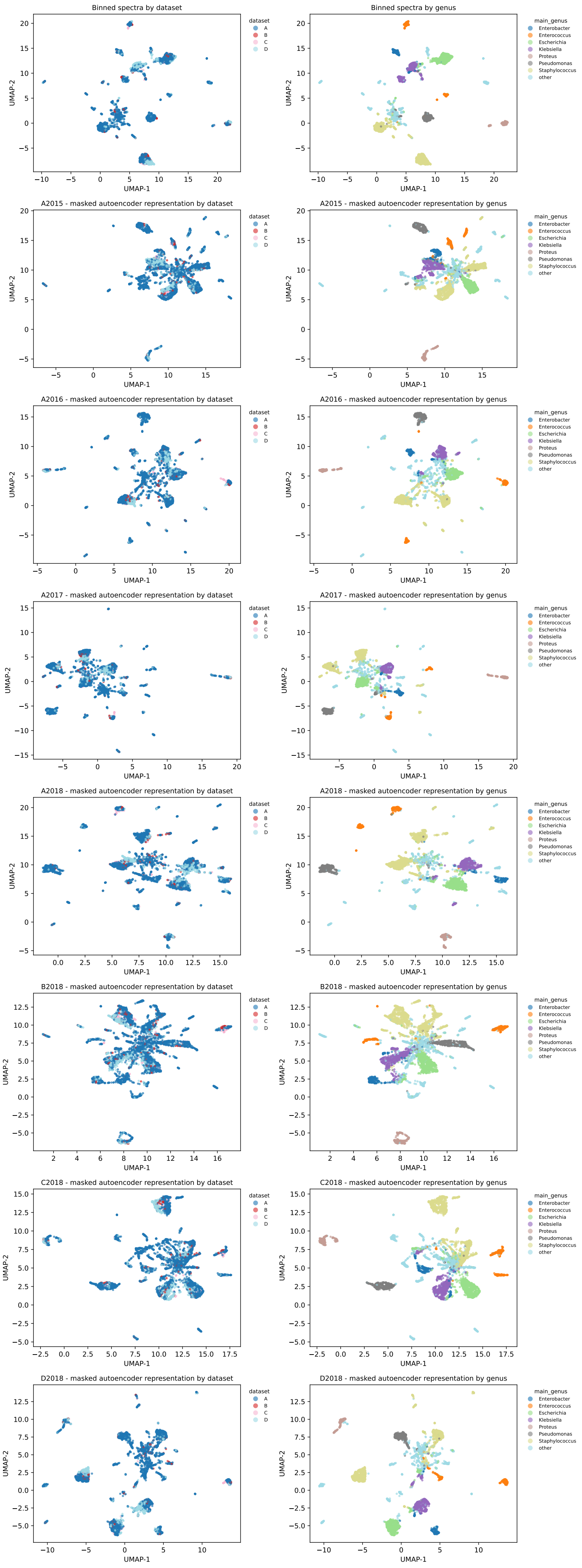

Binned spectra by dataset
Binned spectra by genus
A2015 - masked autoencoder representation by dataset
A2015 - masked autoencoder representation by genus
A2016 - masked autoencoder representation by dataset
A2016 - masked autoencoder representation by genus
A2017 - masked autoencoder representation by dataset
A2017 - masked autoencoder representation by genus
A2018 - masked autoencoder representation by dataset
A2018 - masked autoencoder representation by genus
B2018 - masked autoencoder representation by dataset
B2018 - masked autoencoder representation by genus
C2018 - masked autoencoder representation by dataset
C2018 - masked autoencoder representation by genus
D2018 - masked autoencoder representation by dataset
D2018 - masked autoencoder representation by genus
UMAP-1
UMAP-2
dataset
A
B
C
D
main_genus
Enterobacter
Enterococcus
Escherichia
Klebsiella
Proteus
Pseudomonas
Staphylococcus
other
