# Supplementary material for: Generalizable machine learning models for rapid antimicrobial resistance prediction in unseen health care settings
Source: Gigascience. 2026 Jan 19;15:giaf156. doi: 10.1093/gigascience/giaf156 (PMC12908719; doi:10.1093/gigascience/giaf156)
Supplement: giaf156_GIGA-D-25-00242_original_submission [file giaf156_giga-d-25-00242_original_submission.pdf]

GigaScience

Generalizable machine learning models for rapid antimicrobial resistance prediction in unseen healthcare settings

--Manuscript Draft--

**Manuscript Number:**	GIGA-D-25-00242
**Full Title:**	Generalizable machine learning models for rapid antimicrobial resistance prediction in unseen healthcare settings
**Article Type:**	Research
**Funding Information:**	ETH AI CENTER — Dr. Diane Duroux
**Abstract:**	Background: The deployment of machine learning in clinical settings is often hindered by the limited generalizability of the models. Models that perform well during development tend to underperform in new environments, limiting their clinical utility. This issue affects models designed for the rapid identification of antimicrobial resistance, which is essential to guide treatment decisions. Traditional susceptibility tests can take up to three days, whereas integrating MALDI-TOF mass spectrometry with machine learning has the potential to reduce this to one day. However, model performance declines drastically in hospitals or time frames outside the training data.<br>Results: To improve robustness, we develop advanced feature representations using masked autoencoders (MAE) for MALDI-TOF spectra, and chemical language models and SELF-referencing embedded strings (SELFIES) for antimicrobials. Cross-validated on data from four medical institutions, our models demonstrate improved performance and stability. The MAE and SELFIES encodings increase the area under the precision-recall curve by 6% when evaluated on unseen time periods, while the MAE and Molformer language model encodings improve it by 12% when applied across different hospitals.<br>Conclusions: These results underscore the value of combining deep learning with chemical and spectral information to build generalizable, high-impact clinical AI
**Corresponding Author:**	Diane Duroux<br>ETH Zurich: Eidgenossische Technische Hochschule Zurich<br>Zürich, SWITZERLAND
**Corresponding Author Secondary Information:**	
**Corresponding Author's Institution:**	ETH Zurich: Eidgenossische Technische Hochschule Zurich
**Corresponding Author's Secondary Institution:**	
**First Author:**	Diane Duroux
**First Author Secondary Information:**	
**Order of Authors:**	Diane Duroux
	Paul Philipp Meyer
	Giovanni Visonà
	Niko Beerenwinkel
**Order of Authors Secondary Information:**	
**Additional Information:**	
**Question**	**Response**
Are you submitting this manuscript to a special series or article collection?	No
**Experimental design and statistics**	Yes

Powered by Editorial Manager® and ProduXion Manager® from Aries Systems Corporation

Full details of the experimental design and statistical methods used should be given in the Methods section, as detailed in our Minimum Standards Reporting Checklist. Information essential to interpreting the data presented should be made available in the figure legends.<br><br>Have you included all the information requested in your manuscript?	
**Resources**<br><br>A description of all resources used, including antibodies, cell lines, animals and software tools, with enough information to allow them to be uniquely identified, should be included in the Methods section. Authors are strongly encouraged to cite Research Resource Identifiers (RRIDs) for antibodies, model organisms and tools, where possible.<br><br>Have you included the information requested as detailed in our Minimum Standards Reporting Checklist?	Yes
**Availability of data and materials**<br><br>All datasets and code on which the conclusions of the paper rely must be either included in your submission or deposited in publicly available repositories (where available and ethically appropriate), referencing such data using a unique identifier in the references and in the “Availability of Data and Materials” section of your manuscript.<br><br>Have you have met the above requirement as detailed in our Minimum Standards Reporting Checklist?	Yes
GigaScience has policies and guidelines in place for the use of generative AI-	No

writing tools such as ChatGPT. If you have used such writing tools to assist with writing the manuscript this must be declared and cited in the text. Authors should not list AI-writing tools and other AI-assisted technologies as an author or co-author and should acknowledge that they are fully responsible for text generated or refined by AI-writing tools.<p><br><br>A summary of use (particularly in the introduction or among methods) needs to be included at the end of the paper, and the outputs should also be included as a supplementary file hosted in GigaDB or other open repositories. Please read our guidelines for more information. <p><br><br>By submitting to GigaScience, you are aware of the journal's AI-writing tools policy, and if you have declared use of such tools below, you have acknowledged this where appropriate in your manuscript and have made a summary of use and outputs available. <p><br><b>AI-assisted writing tools have been used in the preparation of this manuscript?	

Manuscript
Click here to access/download;Manuscript;manuscript.pdf

GigaScience, 2025, 1–9

doi: xx.xxxx/xxxx
Manuscript in Preparation
Paper

PAPER

Generalizable machine learning models for rapid antimicrobial resistance prediction in unseen healthcare settings

Diane Duroux[1,2,3,4,*], Paul P. Meyer[5], Giovanni Visonà[6] and Niko Beerenwinkel[2,4]

[1]ETH AI Center, ETH Zurich, Zurich, Switzerland; and [2]Department of Biosystems Science and Engineering, ETH Zurich, Basel, Switzerland; and [3]Department of Quantitative Biomedicine, University of Zurich, Zurich, Switzerland; and [4]SIB Swiss Institute of Bioinformatics, Basel, Switzerland; and [5]Department of Mechanical and Process Engineering, ETH Zurich, Zurich, Switzerland; and [6]Department of Empirical Inference, Max Planck Institute for Intelligent Systems, Tübingen, Germany;

[*]diane.duroux@ai.ethz.ch

Abstract

Background: The deployment of machine learning in clinical settings is often hindered by the limited generalizability of the models. Models that perform well during development tend to underperform in new environments, limiting their clinical utility. This issue affects models designed for the rapid identification of antimicrobial resistance, which is essential to guide treatment decisions. Traditional susceptibility tests can take up to three days, whereas integrating MALDI-TOF mass spectrometry with machine learning has the potential to reduce this to one day. However, model performance declines drastically in hospitals or time frames outside the training data.
Results: To improve robustness, we develop advanced feature representations using masked autoencoders (MAE) for MALDI-TOF spectra, and chemical language models and SELF-referencing embedded strings (SELFIES) for antimicrobials. Cross-validated on data from four medical institutions, our models demonstrate improved performance and stability. The MAE and SELFIES encodings increase the area under the precision-recall curve by 6% when evaluated on unseen time periods, while the MAE and Molformer language model encodings improve it by 12% when applied across different hospitals.
Conclusions: These results underscore the value of combining deep learning with chemical and spectral information to build generalizable, high-impact clinical AI.

Key words: antimicrobial resistance; generalizability; machine learning; representation learning

Introduction

Antimicrobial resistance (AMR) presents a severe threat to public health, with an estimated 1.27 million deaths attributed to bacterial AMR in 2019 [1]. Improved care of severe infections and expanded access to antibiotics could prevent up to 92 million cumulative deaths between 2025 and 2050 [2]. Early identification of AMR is critical to improve the effectiveness of antimicrobial prescriptions and treatment [3]. Recent studies demonstrate that machine learning (ML) models leveraging matrix-assisted laser desorption/ionization time-of-flight (MALDI-TOF) mass spectrometry data of pathogens hold promise by reducing the time to identify antimicrobial resistance from three to one day compared to traditional methods [4, 5, 6]. However, while these models achieve high performance when trained and tested in similar conditions, such as the same hospital and time frame,

Compiled on: June 21, 2025.
Draft manuscript prepared by the author.

the models' accuracy drops when applied to new contexts with (shifted) data from different hospitals or time frames [4]. Such declines in model performance can be particularly hard for the user to detect. The models' predictions can evoke high confidence despite being incorrect, when models are applied to data from an unseen distribution, potentially misleading users and resulting in poor decision-making. Data shift can also introduce biases into model predictions, leading to unfair or inequitable outcomes for certain groups.

Low generalizability across clinical settings, where model performance declines when applied to different hospitals or regions, can stem from factors such as variations in patient demographics, microbial populations, data collection practices, or local healthcare protocols. These contextual differences cause the data distribution in a new location to diverge from that of the training data (i.e., a data shift), resulting in lower model performance. For example, pathogens and resistance mechanisms prevalent in one hospital or region may differ from those in another, complicating accurate AMR prediction across settings. Similarly, low temporal generalizability, reflected as a decrease in model performance when applied to data collected in different years, can arise from evolving microbial populations, changes in resistance mechanisms, or updates in healthcare practices over time. For instance, pathogens and resistance patterns may change over time, posing challenges for models trained on past data to reliably predict AMR in future settings. These temporal data shifts result in decreased model accuracy when applied to more recent data, challenging the ability of models to reliably predict AMR in dynamic clinical environments.

In zero-shot contexts, where hospitals lack the necessary infrastructure or data to develop their own machine learning models, traditional approaches to handling data shifts, such as domain adaptation, fine-tuning, and incorporating a hospital-specific variable, cannot be employed. Instead, data augmentation, continuous monitoring and representation learning can help mitigate the effects of a data shift. Data augmentation enhances generalizability by generating synthetic samples to increase training data diversity. However, designing realistic synthetic data that accurately reflects real-world variability is challenging. Continuous monitoring of model performance is also essential for early detection of a data shift. Regularly evaluating model accuracy allows for timely interventions to maintain performance.

Representation learning could offer a more effective and scalable solution for improving model performance in zero-shot scenarios. It mitigates the impact of limited or imbalanced data, common challenges in healthcare, by leveraging large datasets for pre-training. This process captures diverse patterns, reducing the risk of overfitting when applied to smaller datasets. Additionally, representation learning accommodates evolving data landscapes, as the extracted features should remain relevant even when protocols, equipment, or patient demographics change. In contrast, data augmentation often requires continuous adjustments to address such shifts. Finding learning representations that can be used in different domains is, however, not trivial. It is instead a crucial challenge to face for biomedical applications of machine learning. When data from the target domain is available, methods that rely on active corrections such as transfer learning and domain adaptation [7, 8] offer widely-adopted approaches to improve predictive performance. When no data from the target domain is given, it is still possible to attempt to learn representations that are robust to a domain change, which is the objective of domain generalization [9], a much more challenging and less-established methodology compared to the aforementioned approaches. Therefore, we investigate representation learning as a generalized and scalable approach to deal with data shifts. Representation learning is particularly well-suited to healthcare settings with varying infrastructure, data quality, and privacy constraints.

Efforts to leverage ML for AMR predictions have explored different antimicrobial molecular representations, [10, 11] (i.e., encoding molecules in machine-readable formats) including one-hot encoding, Morgan fingerprints, SMILES strings, DeepSMILES strings with 1D CNN, DeepSMILES strings with transformer, and DeepSMILES strings with recurrent neural network [6, 5]. Experimental results indicate that performance differences associated with different antimicrobial encodings are generally minimal [6]. The authors suggest two potential reasons for this [6]: the training dataset may include too few antimicrobials to learn meaningful relations based on the components of the molecular structure of the antimicrobials, and very similar molecular structures can correspond to vastly different resistance profiles. Richer antimicrobial representations may hold potential to address these two limitations. For example, antimicrobial representations generated by unsupervised transformer-based language models pretrained on a large unlabeled corpus have enabled state-of-the-art results in many downstream, predictive tasks [12, 13]. These representations might include structural relations and highlight relevant differences, enabling models to leverage these broader molecular features for enhanced predictive accuracy. In addition, SELF-referencing embedded strings (SELFIES) [14] have facilitated and streamlined a wide range of applications in chemistry [15] but have not, to our knowledge, been applied in the context of MALDI-TOF-based AMR prediction.

Enriching the representation of pathogens is another promising way to enhance AMR prediction. MALDI-TOF mass spectrometry generates spectra by ionizing pathogen samples with a laser, accelerating the resulting ions through a time-of-flight analyzer, and measuring their mass-to-charge ratios. These spectra serve as unique molecular fingerprints of the pathogens. State-of-the-art AMR prediction models leverage raw MALDI-TOF spectra as representations of the pathogens and projected them to a lower dimensional space. For example, Visona et al. [5] train a classification Multi-Layer Perceptron with Residual Skip-Connections network for AMR prediction. The model first projects the spectra and antimicrobial features to the same dimension before concatenating the two vector representations and using them as the input of a final Multi-Layer Perceptron classifier. De Waele et al.[6], extract spectra representations from a neural network for AMR prediction, with small or medium-sized network variants typically performing best. Emerging techniques, such as masked autoencoders (MAEs) [16], allow to enrich the representation of input data by training the MAEs to reconstruct missing information. Using a MAE could enable the model to construct richer spectra representations, which capture essential patterns and reduce noise.

This study aims to develop a novel model architecture to improve generalizability, reducing the need to develop separate models for each hospital or data collection year. We quantify the generalizability of AMR prediction models and examine the impact of different encodings on model performance in a zero-shot setting. Additionally, we assess how these encodings influence model stability when transitioning from a non-zero-shot to a zero-shot context. By addressing limitations in generalizability across clinical settings and years, our work aims to enhance predictive accuracy and contribute to more adaptable and reliable AMR diagnostics.

Methods

Problem Definition and Notation

We denote the MALDI-TOF mass spectra of pathogens by $\mathbf{X}_s \in \mathbb{R}^{N_s \times D_s}$, where N_s is the number of pathogen spectra samples, and D_s is the dimensionality of each spectrum. Antimicrobial drugs are represented by $\mathbf{X}_d \in \mathbb{R}^{N_d \times D_d}$, where N_d is the number of drugs, and D_d is the dimensionality of each antimicrobial molecular

representation. For each pathogen-antimicrobial combination with an available susceptibility test outcome, a binary label $y \in \{0, 1\}$ is provided, where 0 indicates sensitivity and 1 indicates resistance. To predict resistance versus susceptibility, we learn a function that maps each pair consisting of pathogen i and antimicrobial j to a binary label, $f(\mathbf{X}_s^i, \mathbf{X}_d^j) =: \hat{y}_{ij}$, approximating the true relationship. The primary objective of this study is to improve the representations of both input data types—mass spectra of pathogens and antimicrobial profiles—in order to improve the prediction of resistance or sensitivity y of each pathogen to a given antimicrobial (Fig. 1). In particular, we aim to make the model more adaptable to new contexts, such as different data collection years or hospitals.

Data and pre-processing

The publicly available DRIAMS dataset [17] consists of MALDI-TOF mass spectrometry data collected from patients in the Swiss healthcare system. This dataset includes data from four distinct diagnostic laboratories, collected from 2015 to 2018: University Hospital Basel (designated as DRIAMS-A), Cantonal Hospital Basel-Land (DRIAMS-B), Cantonal Hospital Aarau (DRIAMS-C), and the Viollier laboratory service provider (DRIAMS-D). Data bias exists within DRIAMS-A due to one particular workstation (HospitalHygene), where samples were captured using a different medium and skewed heavily toward resistant samples. Following Weis et al. [4], we excluded data from this workstation to mitigate potential biases.

The filtered dataset encompasses 54,283 unique mass spectra associated with 631,167 antimicrobial resistance phenotypes. It covers 65 antimicrobials. Each entry provides a pathogen mass spectrum derived from a patient sample, with annotations indicating susceptibility or resistance to antimicrobials. We used the 6000-dimensional binned mass spectra vector representation, consistent with the approach of Weis et al. [4].

The dataset has a noticeable imbalance in the number of samples according to the hospital of origin (Suppl. Table 1). Specifically, A2017 has a substantially higher number of samples (179,334) compared to all other entries. Hospitals B, C, and hospital A with data collected in 2015 have fewer samples (32,377, 47,586, and 11,610, respectively).

Mass spectra encoding

We compare two pathogen representations: binned MALDI-TOF mass spectra and encodings generated by masked autoencoders (MAEs). The spectra were binned using a bin size of 3 m/z units, effectively reducing dimensionality while preserving spectral patterns. This bin size is sufficiently small to separate mass peaks, while still being large enough to maintain computational efficiency [4].

We used a MAE to generate novel encodings of mass spectra for downstream classification tasks. This unsupervised approach involves masking out random bins in each spectrum and training the model to reconstruct the missing information. For each training spectrum, multiple masked copies are generated. By creating varied versions of each spectrum, we multiply the training data and expose the model to a variety of masked versions of each training sample. This variety may help the autoencoder learn resilient patterns by repeatedly encountering and reconstructing diverse masked inputs.

Let $\mathbf{X}_s^{\text{masked}}$ represents the spectra data after random masking is applied. The training dataset consists of these masked spectra paired with their corresponding original versions, serving as reconstruction targets. The feedforward MAE comprises two components:

- The encoder, $f_{\text{MAE-Enc}} : \mathbb{R}^{D_s} \to \mathbb{R}^{H_s}$, is a single dense layer with 512 neurons and ReLU activation. It maps the masked spectra into a low-dimensional latent space of dimension H_s, learning a compact representation $\mathbf{z}_s$.
- The decoder, $f_{\text{MAE-Dec}} : \mathbb{R}^{H_s} \to \mathbb{R}^{D_s}$, reconstructs the original spectra from the encoded representation using a dense layer with sigmoid activation, training the model to predict missing values and capture comprehensive patterns.

After performing a parameter search (Suppl. Table 4), the optimal parameters were selected as follows: the number of copies per pathogen sample was set to 10, the mask ratio ranged from 0.2 to 0.5, the encoding dimension was 512, the number of epochs was 100, the batch size was 50, and the learning rate was 0.001.

Throughout training, the model learns to reconstruct the original spectra from masked inputs, prompting the encoder to develop a robust and generalizable feature representation. Once training is complete, we use the encoder to extract the encoded representations by processing the original, unmasked spectra data. The spectrum representation $\mathbf{z}_s$ for a given pathogen is $\mathbf{z}_s = f_{\text{MAE-Enc}}(\mathbf{X}_s)$. This final representation serves as the input for downstream tasks.

Antimicrobial encoding

We compare three types of antimicrobial representations: traditional molecular fingerprints, encodings generated by Molformer transformer-based model for molecular representation learning [12], and SELF-referencing embedded strings (Selfies) [14].

Molecular fingerprinting [18] is a widely used technique in chemical informatics and antimicrobial discovery, converting molecular structures into numerical features for use in machine learning models. Fingerprints represent a molecule as a binary vector, where each bit indicates the presence or absence of specific substructures or chemical patterns. This approach captures different aspects of a molecule's structure, including topological, physio-chemical, and other structural properties, making it highly effective in various applications [19, 20, 21]. In Visona et al. [5], three standard fingerprinting techniques were evaluated: the molecular ACCess systems (MACCS) keys (166 bits) [22], the PubChem fingerprints (PubChemFP) (881 bits) [23], and the 1024-bit Morgan fingerprints (1024 bits) [24]. No single fingerprint class demonstrated consistent superiority over the others. The authors opted to use Morgan fingerprints due to their widespread adoption in small molecule screening and proven robust performance across various tasks. Following this approach, we also use 1024-bit Morgan fingerprints.

To capture more complex features of antimicrobials, we leverage Molformer [12], a large-scale transformer model pre-trained for molecular representation learning. Unlike traditional fingerprinting, Molformer encodings are learned representations that aim to capture semantic relationships within molecular structures beyond discrete substructures. Molformer applies self-attention mechanisms across molecular graphs, allowing it to model interactions and dependencies among atoms and bonds. This structure may help capture features of molecular conformation, functional groups, and other chemical characteristics, providing a more comprehensive molecular encoding. Specifically, we used the available model that is pre-trained on 10% of the Zinc and PubChem database. Each antimicrobial is encoded as a vector of dimension 768.

We also evaluate SELF-referencing embedded strings (SELFIES) [14], a string-based representation for molecules. This approach addresses a critical limitation of the standard molecular representation, SMILES [25], which often produces strings that do not correspond to valid molecules. In contrast, SELFIES overcomes this issue by ensuring that every SELFIES

string represents a valid molecule and that every molecule can be represented using SELFIES. Starting with the SMILES representation of the antimicrobials in the DRIAMS dataset, we convert the SMILES strings into SELFIES. To ensure uniform encoding dimensions, we transform the SELFIES strings into one-hot encodings by first constructing an alphabet from the SELFIES strings. Each SELFIES string is then converted into a padded one-hot encoding, which is subsequently flattened. This process results in each antimicrobial being represented as a vector of dimension 24,160. We define the antimicrobial encoder (Molformer or SELFIES) as $f_{\text{antimicrobial-Enc}} : \mathbb{R}^{D_d} \to \mathbb{R}^{H_d}$, where H_d is the hidden dimensionality of the antimicrobial representation. The antimicrobial representation $\mathbf{z}_d$ for a given antimicrobial is the image $\mathbf{z}_d = f_{\text{antimicrobial-Enc}}(\mathbf{X}_d)$.

Model architecture

Since H_s and H_d differ, we introduce two projection layers: a projection for the mass spectrum encoding: $h_s : \mathbb{R}^{H_s} \to \mathbb{R}^H$, and a projection for the antimicrobial encoding: $h_d : \mathbb{R}^{H_d} \to \mathbb{R}^H$. The projected encodings are then: $\mathbf{z}'_s = h_s(\mathbf{z}_s) \in \mathbb{R}^H$ and $\mathbf{z}'_d = h_d(\mathbf{z}_d) \in \mathbb{R}^H$. After projecting both representations to the same dimensionality H, we concatenate $\mathbf{z}'_s$ and $\mathbf{z}'_d$ to form a combined representation $\mathbf{z}_{sd} = (\mathbf{z}'_s, \mathbf{z}'_d) \in \mathbb{R}^{2H}$

In Visona et al. [5], three primary models are explored: (1) encodings derived by applying principal component analysis to mass spectra and chemical fingerprints, which are concatenated and used as input for a logistic regression model; (2) joint representations generated by Siamese networks, subsequently utilized as input for logistic regression to predict resistance; and (3) a classification Multi-Layer Perceptron (MLP) with Residual Skip-Connections. Building on the findings of Visona et al. [5], which identified the MLP with Residual Skip-Connections [26] as achieving the highest performance, we trained this network to predict the probability of resistance for antimicrobial-spectrum pairs. The concatenated vector $\mathbf{z}_{sd}$ is used as input to a ResMLP model to classify $\mathbf{z}_{sd}$ into resistant or sensitive categories. $f_{\text{ResMLP}} : \mathbb{R}^{2H} \to \{0, 1\}$ for classification $y' = f_{\text{ResMLP}}(\mathbf{z}_{sd})$. This model incorporates skip connections, which provide a path for the gradients of the loss with respect to the model weight matrices to bypass certain layers during back-propagation and reach deeper layers in the network, typically enhancing the training process [27].

The final prediction pipeline is as follows (Fig. 1): (1) Encode the pathogen spectrum: $\mathbf{z}_s = f_{\text{MAE-Enc}}(\mathbf{X}_s)$. (2) Encode the antimicrobial: $\mathbf{z}_d = f_{\text{antimicrobial-Enc}}(\mathbf{X}_d)$. (3) Project both encodings and concatenate the projections: $\mathbf{z}'_s = h_s(\mathbf{z}_s)$, $\mathbf{z}'_d = h_d(\mathbf{z}_d)$, and $\mathbf{z}_{sd} = (\mathbf{z}'_s, \mathbf{z}'_d)$. (4) Classify with ResMLP: $y' = f_{\text{ResMLP}}(\mathbf{z}_{sd})$.

Performance assessment

We compared the baseline antimicrobial resistance model, which uses as input a 6,000-dimensional binned MALDI-TOF mass spectra vector for pathogen representation and Morgan fingerprints for antimicrobial encoding, to the competitive approaches outlined in Section . In total, we compare six input configurations, combining three antimicrobial representations (Morgan fingerprints, MoLFormer, and SELFIES) with two spectra encodings (binned MALDI-TOF and masked autoencoder-based representations).

We designed two data splits to reflect different data-generating processes and examine the prediction capabilities of the previously described machine learning models. Notably, to prevent data leakage, each data split was designed so that no pathogen appeared in both the training and test sets.

i. *Hospital zero-shot split*: Select data from the DRIAMS dataset collected in 2018. Let $i, j \in \{A, B, C, D\}$ represent the hospitals in the dataset. Train the model on data from hospital i. Test the model on data from hospital j, where $j \neq i$.
ii. *Year zero-shot split*: Select data from the DRIAMS A. Let i, $j \in \{2015, 2016, 2017, 2018\}$ represent the year of data collection. Train the model on data from year i. Test the model on data from year j, where $j \neq i$.

We report three standard classification metrics for imbalanced data: area under the precision-recall curve (AUPRC), balanced accuracy, and Matthews Correlation Coefficient (MCC).

Results

We illustrate the generalizability challenges of the baseline antimicrobial resistance model [5], which utilizes a 6000-dimensional binned MALDI-TOF mass spectra vector representation for pathogens and Morgan fingerprints for antimicrobial representations. We then investigate how alternative representations of both MALDI-TOF spectra and antimicrobials affect zero-shot prediction performance, and evaluate whether the most effective encodings lead to improved model stability when transitioning from non-zero-shot to zero-shot settings (Fig. 1).

Baseline AMR model performance substantially declines in zero-shot scenarios

The baseline model achieves the highest AUPRC values when trained and tested on data collected within the same year (Fig. 2 **a**), with AUPRC scores ranging from 0.60 for 2015 data to 0.75 for samples from 2017, indicating stable performance in a consistent temporal context. However, performance declines when the model is applied across different years, highlighting its sensitivity to temporal variations. On average, AUPRC decreases by 0.22 when tested on 2015 samples using models trained on data from other years. For 2016, the average decrease is 0.16; for 2017, it is 0.08; and for 2018, the decrease is 0.11.

A similar pattern emerges with spatial generalizability, though the effect is even more pronounced. The model demonstrates robust performance when trained and tested on data from the same hospital, achieving AUPRC values ranging from 0.60 for hospital D to 0.81 for hospital C. However, performance drops when the model is trained on data from one hospital and tested on another, with AUPRC values consistently falling below 0.44. On average, the AUPRC decreases by 0.38 when tested on data from Hospital A (2018) and trained on data from other years. For Hospital B, the average decrease is 0.33; for Hospital C, it is also 0.33; and for Hospital D, the decrease is 0.45.

Overall, these findings highlight the challenges the AMR model faces in adapting across different hospitals and years. Additional plots for MCC and balanced accuracy are available in the supplementary materials.

Advanced feature representation boosts model performance in year and hospital zero-shot contexts

We analyze how various encoding methods impact model performance when no hospital-specific or year-specific data are available for training (zero-shot setting). We evaluate how well different encodings enable generalization to unseen hospitals or unseen years of data collection. We illustrate the generalizability of the model across clinical settings and years, measured by the average AUPRC across training sets in Fig. 3. The baseline corresponds to the light blue bars. Since the AUPRC of a random classifier is equal to the proportion of the positive class in the data

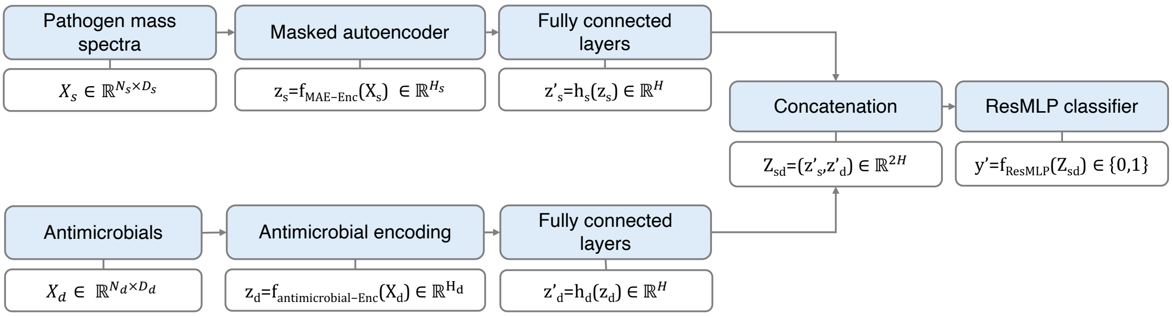

Fig. 1. Model architecture: A masked autoencoder is applied to the binned representation of pathogen MALDI TOF mass spectra ($D_S = 6000$, and $H_S = 512$). Antimicrobials are encoded using the Molformer transformer ($H_{d,\text{Molformer}} = 768$) or SELFIES ($H_{d,\text{SELFIES}} = 24160$). The pathogen and antimicrobial encodings are projected into a common latent space. These encodings are then concatenated to form a unified feature vector, which serves as input to a ResMLP classifier for final prediction.

set, we indicate the proportion of resistant labels in each test set for reference. It ranges from 0.15 for D2018 to 0.25 for C2018.

Fig. 3 **a** presents the AUPRC of the model when trained on data from specific years and tested on datasets from different years. The test datasets correspond to hospital A, with samples collected in 2015, 2016, 2017, and 2018. Across all test cases, the baseline is consistently outperformed using alternative encodings, with the most substantial improvement attributed to the new pathogen representation. Replacing binned spectra with MAE encodings enhances AUPRC by 4% on average across the four test cases. MAE encodings also tend to reduce variability in model performance across different training sets, particularly for A2016, A2017, and A2018. Incorporating an advanced antimicrobial representation alongside MAE encodings further improves performance. Among antimicrobial representations, Molformer encodings outperform Morgan fingerprints for three test sets, although the difference is small. SELFIES representations consistently achieve the highest AUPRC scores. Overall, using MAE encodings for pathogen representation and SELFIES representations for antimicrobial enhanced year-to-year generalizability. In our dataset, this approach led to an average performance increase of 6% (55.3 to 61.7) compared to the baseline.

Fig. 3 **b** presents the AUPRC of the model when tested on samples from a hospital not included in the training set. The hospital generalizability task is inherently more challenging than the temporal generalizability task, as evidenced by the decline in AUPRC between Figure 3 **a** and **b**. Despite this challenge, several alternative encoding techniques outperformed the baseline, primarily due to the MAE encodings of the pathogens. Replacing binned spectra with MAE encodings led to an average AUPRC increase of 10% across the four test cases. This improvement is more pronounced in the hospital generalization setting compared to the year-to-year generalization scenario. In the hospital generalization task, the most effective antimicrobial representations were Molformer encodings closely followed by Morgan fingerprints. Overall, using MAE encodings for pathogen representation combined with Molformer encodings or Morgan fingerprints for antimicrobial enhanced hospital-to-hospital generalizability, with an absolute increase in AUPRC of 12% (33.7 to 45.2) and 11% (33.7 to 44.5) respectively, compared to the baseline.

Masked autoencoder pathogen encodings increase the non-zero-shot to zero-shot stability

This section examines *non-zero-shot to zero-shot (NS-ZS) stability*, i.e., the differences in model performance when transitioning from a non-zero-shot to a zero-shot setting. We explore how encoding choices influence the gap between these two settings, highlighting which methods improve stability across different data

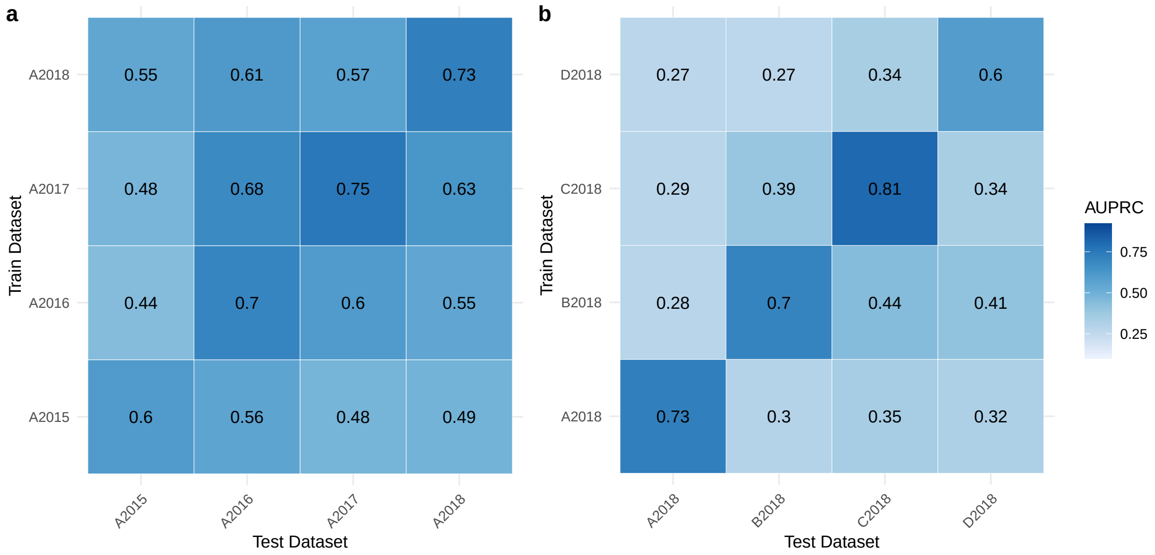

Fig. 2. AUPRC across (a) years of data collection or (b) hospitals, with pathogens represented with 6000-dimensional binned MALDI TOF mass spectra vectors and antimicrobials represented with Morgan fingerprints. For reference, the resistant-to-susceptible class ratio is 0.19 for A2015, 0.23 for A2016, 0.19 for A2018, 0.17 for A2018 and B2018, 0.25 for C2018, and 0.15 for D2018.

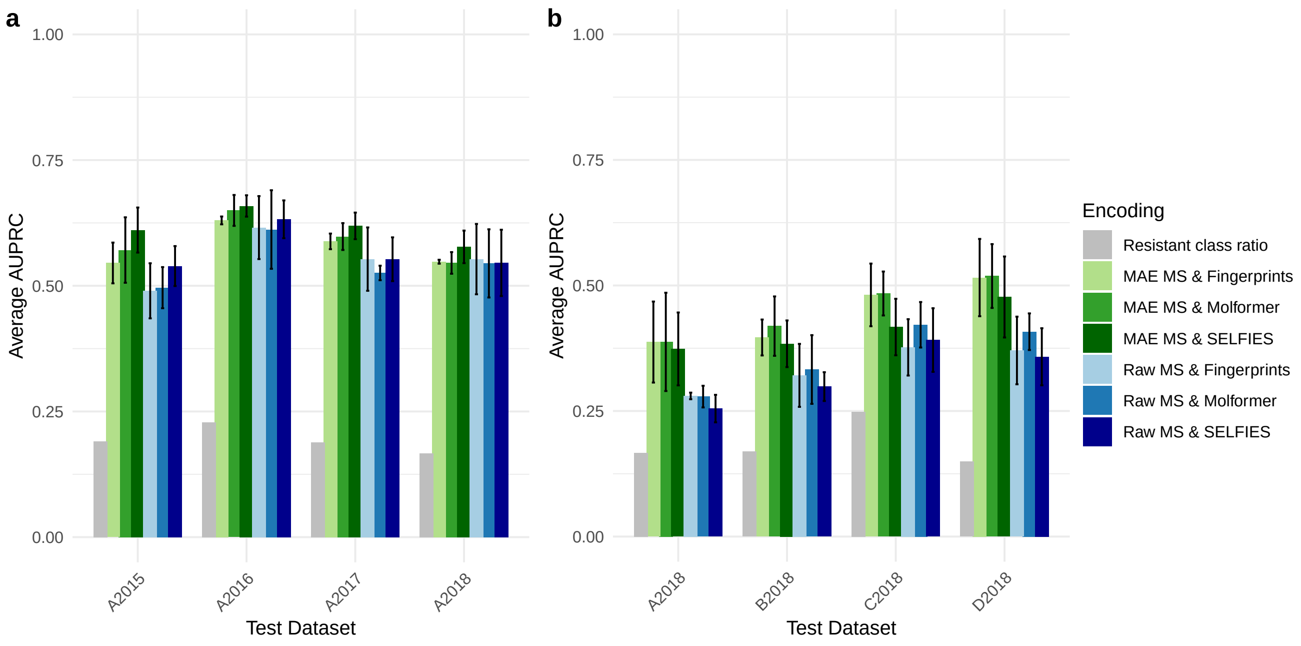

Fig. 3. Average AUPRC across different test sets for (a) year-zero-shot (b) hospital-zero-shot analyses. Error bars represent standard deviation across training sets.

availability scenarios. Assessing this NS-ZS stability will provide insights into the reliability of predictions in unseen contexts. Figure 4 illustrates the NS-ZS stability by showing performance decrease when transitioning from a non-zero-shot-context (model trained and tested on i) prediction scenario to a zero-shot scenario (model trained on i and tested on j, $j \neq i$). A smaller average decrease in AUPRC suggests a more stable model, indicating better generalizability across different contexts. The exact values and standard deviations obtained across training sets are available in Supplementary Tables 2 and 3.

The MAE encodings of the pathogen drive the highest improvement in model NS-ZS stability across time (Figure 4 **a**). On average, using MAE encoding increases the difference in AUPRC by 4% compared to raw binned spectra. When ranking the AUPRC difference for each test set and averaging these ranks, the top-performing encoding is MAE mass spectra combined with SELFIES antimicrobial encoding, with an average rank of 2. It is followed by MAE-Molformer, MAE-fingerprints, raw mass spectra-Molformer, raw mass spectra-SELFIES, and raw mass spectra-fingerprints. Thus, we identify MAE mass spectra combined with SELFIES antimicrobial encoding as the most stable configuration across temporal contexts. This approach reduces the NS-ZS mean AUPRC decrease from 14% with the baseline to 8%, highlighting its effectiveness in enhancing generalizability.

Hospital NS-ZS stability remains a more complex challenge, as reflected in the larger absolute AUPRC difference between Figure 4 **a** (temporal NS-ZS stability) and Figure 4 **b** (hospital NS-ZS stability). In the hospital zero-shot scenario, we observe the same overall trend as in the temporal zero-shot scenario, where the main improvement in model NS-ZS stability is driven by MAE encodings of the pathogen—but to an even greater extent. On average, using MAE encoding increases the NS-ZS difference in AUPRC by 8% compared to raw binned spectra. When ranking the AUPRC difference across test sets and averaging these ranks, the top-performing encoding in this setting is MAE mass spectra combined with Morgan fingerprints, followed by MAE-Molformer, MAE-SELFIES, raw mass spectra-Molformer, raw mass spectra-fingerprints, and raw mass spectra-SELFIES. Thus, we identify MAE mass spectra combined with Morgan fingerprints as the most stable configuration across hospitals. This approach reduces the mean AUPRC decrease from 35% with the baseline to 28%.

Maximizing generalizability by increasing variability and sample size in training sets

We compare the impact of training a model with data from one hospital versus three hospitals in the hospital zero-shot scenario. In the following, we focus on the combination of MAE & Molformer encodings and MAE & Morgan fingerprint encodings, as these contribute the most to improving generalizability (Sections and). Training on data from three hospitals not only increases the sample size but also introduces greater variability in the training set. In clinical decision support tools, aggregating data from multiple hospitals could be detrimental due to excessive data shifts. This issue arises in the AMR prediction task in the non-zero-shot (NS) scenario, where the test samples come from a hospital included in the training set. For instance, when using the MAE for pathogen encoding, training on a single hospital can be more beneficial than using data from three hospitals (Suppl. Fig. 5).

In the hospital zero-shot (ZS) scenario, this pattern does not hold. Instead, incorporating data from three hospitals consistently improves model performance compared to using data from one hospital (Fig. 5). On average, across four test hospitals, training with three hospitals enhances AUPRC performance by 13% when using baseline pathogen and antimicrobial encoding. Notably, this gain from increased data variability slightly exceeds the 12% improvement achieved by the best performing encoding (MAE & Molformer). Additionally, combining data from multiple hospitals with robust encodings (MAE & Molformer) further enhances the model's generalizability, yielding an average AUPRC increase of 14%.

When training the model with the baseline encodings on only one external hospital (ZS), the AUPRC decreased by 35% compared to training and testing on the same hospital data (NS). Incorporating data from three external hospitals along with robust encodings reduces the AUPRC decrease to 21%. In the ZS experiments, the MAE & Molformer combination achieved the highest scores (Fig. 5). This encoding combination also provided

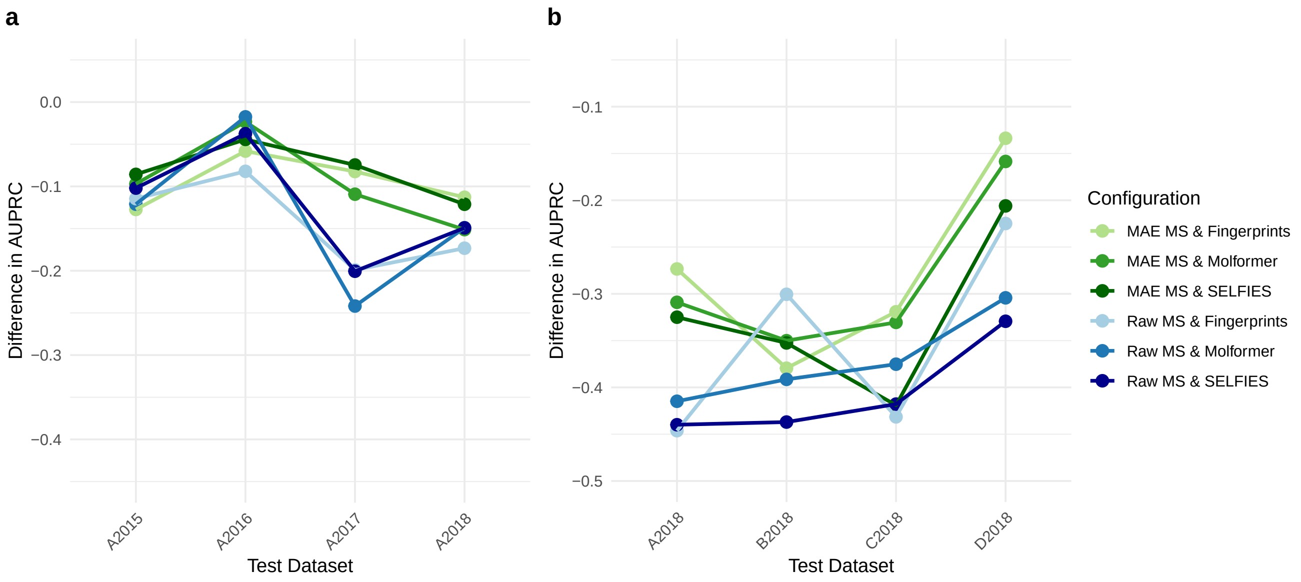

Fig. 4. Zero-shot to non-zero-shot model stability: difference in AUPRC between performance obtained when the model is trained and tested on the same context, i.e., (a) year and (b) hospital, versus trained and tested on difference contexts.

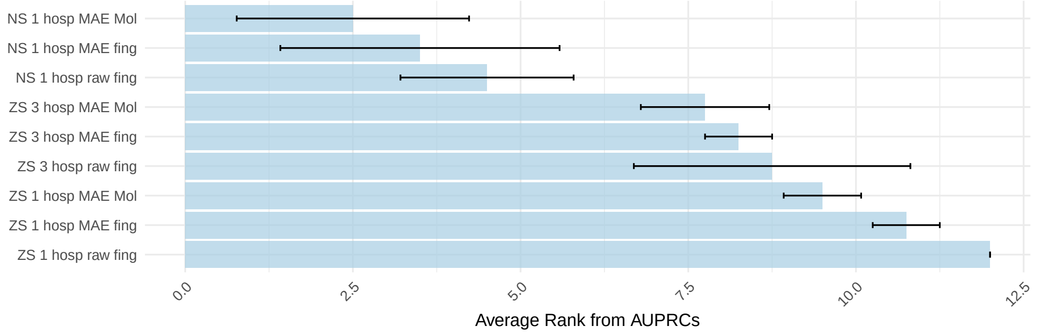

Fig. 5. Average ranks across four test hospitals (A, B, C, and D) based on AUPRC. Non-zero shot (NS) means the test hospital was part of the training set, while hospitals in training and testing are different for the zero-shot (ZS) scenario. The confidence intervals show the standard deviation across the four test hospitals. Training was done using either one or three hospitals. When only one hospital was used in the ZS setting, AUPRC ranks were averaged over three models (e.g., for test hospital D, models trained on A, B, and C were averaged). Similarly, in the NS setting with three hospitals, ranks were averaged over three models trained on different hospital combinations (e.g., ABD, BCD, and ACD for test hospital D).

the greatest improvement in the NS one-hospital scenario. Overall, these findings highlight the importance of integrating robust encodings with data from multiple hospitals, whenever possible, to maximize model generalizability and performance in zero-shot contexts.

Discussion

In this study, we aimed to enhance the generalizability of models to eliminate the need for developing a separate model for each hospital or each year of data collection. To overcome this challenge, we focus on creating robust input features that enhance the generalizability of models across different hospitals and time. In particular, we investigate the use of advanced representations for both pathogens and antimicrobials to improve the AMR model properties in zero-shot scenarios. Using masked autoencoders encodings for pathogens and SELFIES or transformer models for antimicrobials, we evaluate not only the overall improvement in predictive performance but also the enhanced stability of the models across different contexts. Our findings demonstrate that these alternative encodings outperform state-of-the-art methods for AMR prediction, offering a more robust and generalizable approach.

Strategies for antimicrobial encoding. We utilized the pre-trained Molformer transformer model to generate antimicrobial encodings due to its strong performance on classification and regression benchmarks from MoleculeNet [28]. We did not anticipate that fine-tuning Molformer would significantly improve results because the classifier had already encountered most antimicrobials or very similar ones during training. Furthermore, we used checkpoints from a Molformer model pre-trained on a dataset of approximately 100 million molecules. This dataset consisted of 10% of molecules from ZINC and 10% from PubChem, originally used for training the Molformer-XL model. Access to a Molformer model trained on the full 100% dataset, rather than just a 10% subset, could potentially lead to further improvements in performance by providing more comprehensive encodings.

Incorporating hospital-specific context. To improve the applicability of models across different hospitals, an additional step would be to identify factors that contribute to performance degradation across hospitals. Examples of such factors could include the type of MALDI-TOF machine used, the sample material (e.g., blood, urine, or tissue), the type of agar plate used for culturing before MALDI-TOF MS analysis, or the preprocessing techniques employed in different laboratories. By including these

contextual variables as additional features in the model, we could mitigate the variability in methodologies and infrastructure, thus improving generalizability. Training on a larger and more diverse dataset would improve the model's robustness and enhance its ability to generalize across different environments. Unfortunately, these types of metadata are currently not available in publicly accessible datasets.

Alternative strategies to handle data shift. In this work, we address the challenge faced by hospitals that lack the necessary infrastructure or data to develop their own machine learning models. Nevertheless, if hospitals had access to sufficient data and computational resources, alternative strategies could be employed to mitigate the effects of a data shift, including domain adaptation, fine-tuning, and incorporating hospital-specific variables. Domain adaptation techniques aim to align the distributions of source and target domains by minimizing discrepancies between them. This can be achieved through methods such as adversarial training, where a model learns to make predictions while an adversarial component ensures that source and target features become indistinguishable, or importance weighting, which adjusts the influence of source samples based on their relevance to the target domain. Fine-tuning involves training a model on data from one hospital and refining it with a small set of observations from a new hospital. This helps adapt the model to local variations. Another strategy is to incorporate a variable representing the hospital itself into the model, allowing it to account for institution-specific differences. While these approaches can improve model performance, they come with logistical challenges, such as requiring access to target domain data, which is often restricted in healthcare due to privacy constraints. Still, if access to new hospital data were available, these methods could be combined with representation learning to further enhance performance.

Limitations of the study. The approach presented has several additional limitations. First, the use of alternative encodings, such as MAE, Molformer, or SELFIES, reduces the interpretability of the results. For example, when using MALDI-TOF MS data, it is possible to identify specific features (m/z units) that contribute most to antimicrobial resistance prediction. This, in turn, can provide insights into which genes may be involved in resistance mechanisms. Similarly, Morgan fingerprints can be traced back to specific chemical substructures, allowing for a more interpretable link between molecular structure and model predictions. These interpretability advantages are diminished when using the more abstract encodings. Second, the use of MALDI-TOF mass spectrometry inherently limits resistance detection to mechanisms that manifest as changes in the spectral profile. For example, resistance mechanisms involving protein expression changes may be detectable, while those relying solely on point mutations might go unnoticed. Moreover, while we evaluated our approach on data from four different hospitals, these institutions are geographically close (northern switzerland). As a result, the models may still benefit from regional similarities. Applying the models to hospitals in more distant or diverse geographic locations could reveal additional generalizability challenges.

Clinical deployment. While the antimicrobial resistance model demonstrates promising performance across multiple hospitals, it is not yet ready for direct clinical deployment. Beyond legal and regulatory considerations, several key steps remain before it can be integrated into clinical practice. First, model performance could likely be improved by incorporating additional patient-level features that are routinely available to clinicians, such as prior antibiotic use, recent hospitalization, immunosuppression, or travel history. These factors may influence the risk of antimicrobial resistance and could enhance both the accuracy and clinical relevance of the model. Second, it is important to evaluate the model's role within the broader context of antimicrobial stewardship. In particular, we should assess whether the model could support clinical decisions such as the de-escalation of broad-spectrum antibiotics in favor of narrower, more targeted therapies. Third, prospective validation in real-world clinical settings is necessary to assess the model's robustness and generalizability beyond retrospective data. Addressing these aspects will be essential for transitioning from a proof-of-concept model to one that is clinically trustworthy and actionable.

Availability of Source Code and Requirements

Project name: ZeroShotAMR
Project home page: https://github.com/DianeDuroux/ZeroShotAMR
Operating system(s): Platform independent
Programming language: python
Other requirements: pytorch_lightning, scikit_learn, shap, tensorflow, torch, tqdm
License: GNU GPL v3.0

Data Availability

The MALDI-TOF spectra used in this project can be obtained by downloading the DRIAMS dataset from https://datadryad.org/stash/dataset/doi:10.5061/dryad.bzkh1899q [17].

Conflict of interest

None declared.

Acknowledgments

This research was primarily supported by the ETH AI Center.

Author contributions

DD conceptualized the project. DD and GV contributed to the design of the methodology. DD and PPM were responsible for programming, software development, and performing the analyses. All authors participated in the interpretation of the data. DD wrote the original draft, and all authors reviewed and approved the final manuscript.

References

1. Murray CJ, Ikuta KS, Sharara F, Swetschinski L, Aguilar GR, Gray A, et al. Global burden of bacterial antimicrobial resistance in 2019: a systematic analysis. The lancet 2022;399(10325):629–655.
2. Naghavi M, Vollset SE, Ikuta KS, Swetschinski LR, Gray AP, Wool EE, et al. Global burden of bacterial antimicrobial resistance 1990–2021: a systematic analysis with forecasts to 2050. The Lancet 2024;404(10459):1199–1226.
3. Aljeldah MM. Antimicrobial resistance and its spread is a global threat. Antibiotics 2022;11(8):1082.
4. Weis C, Cuénod A, Rieck B, Dubuis O, Graf S, Lang C, et al. Direct antimicrobial resistance prediction from clinical MALDI-TOF mass spectra using machine learning. Nature Medicine 2022;28(1):164–174.
5. Visonà G, Duroux D, Miranda L, Sükei E, Li Y, Borgwardt K, et al. Multimodal learning in clinical proteomics: enhancing

antimicrobial resistance prediction models with chemical information. Bioinformatics 2023;39(12):btad717.
6. De Waele G, Waegeman W, Menschaert G. An antimicrobial drug recommender system using MALDI-TOF MS and dual-branch neural networks. bioRxiv 2023;p. 2023–09.
7. Zhuang F, Qi Z, Duan K, Xi D, Zhu Y, Zhu H, et al. A comprehensive survey on transfer learning. Proceedings of the IEEE 2020;109(1):43–76.
8. Farahani A, Voghoei S, Rasheed K, Arabnia HR. A brief review of domain adaptation. Advances in data science and information engineering: proceedings from ICDATA 2020 and IKE 2020 2021;p. 877–894.
9. Wang J, Lan C, Liu C, Ouyang Y, Qin T, Lu W, et al. Generalizing to unseen domains: A survey on domain generalization. IEEE transactions on knowledge and data engineering 2022;35(8):8052–8072.
10. David L, Thakkar A, Mercado R, Engkvist O. Molecular representations in AI-driven drug discovery: a review and practical guide. Journal of cheminformatics 2020;12(1):56.
11. McGibbon M, Shave S, Dong J, Gao Y, Houston DR, Xie J, et al. From intuition to AI: evolution of small molecule representations in drug discovery. Briefings in bioinformatics 2024;25(1):bbad422.
12. Ross J, Belgodere B, Chenthamarakshan V, Padhi I, Mroueh Y, Das P. Large-scale chemical language representations capture molecular structure and properties. Nature Machine Intelligence 2022;4(12):1256–1264.
13. Irwin R, Dimitriadis S, He J, Bjerrum EJ. Chemformer: a pre-trained transformer for computational chemistry. Machine Learning: Science and Technology 2022;3(1):015022.
14. Krenn M, Häse F, Nigam A, Friederich P, Aspuru-Guzik A. Self-referencing embedded strings (SELFIES): A 100% robust molecular string representation. Machine Learning: Science and Technology 2020;1(4):045024.
15. Krenn M, Ai Q, Barthel S, Carson N, Frei A, Frey NC, et al. SELFIES and the future of molecular string representations. Patterns 2022;3(10).
16. He K, Chen X, Xie S, Li Y, Dollár P, Girshick R. Masked autoencoders are scalable vision learners. In: Proceedings of the IEEE/CVF conference on computer vision and pattern recognition; 2022. p. 16000–16009.
17. Weis C, al. DRIAMS: Database of Resistance Information on Antimicrobials and MALDI-TOF Mass Spectra. Dryad 2022;.
18. Rogers D, Hahn M. Extended-connectivity fingerprints. Journal of chemical information and modeling 2010;50(5):742–754.
19. Yang J, Cai Y, Zhao K, Xie H, Chen X. Concepts and applications of chemical fingerprint for hit and lead screening. Drug Discovery Today 2022;27(11):103356.
20. Ran B, Chen L, Li M, Han Y, Dai Q. Drug-Drug Interactions Prediction Using Fingerprint Only. Computational and Mathematical Methods in Medicine 2022;2022(1):7818480.
21. Li Y, Huang YA, You ZH, Li LP, Wang Z. Drug-target interaction prediction based on drug fingerprint information and protein sequence. Molecules 2019;24(16):2999.
22. Durant JL, Leland BA, Henry DR, Nourse JG. Reoptimization of MDL keys for use in drug discovery. Journal of Chemical Information and Computer Sciences 2002;42(6):1273–1280.
23. Wang Y, Bryant SH, Cheng T, Wang J, Gindulyte A, Shoemaker BA, et al. Pubchem bioassay: 2017 update. Nucleic Acids Research 2017;45(D1):D955–D963.
24. Morgan HL. The generation of a unique machine description for chemical structures-a technique developed at chemical abstracts service. Journal of Chemical Documentation 1965;5(2):107–113.
25. Weininger D. SMILES, a chemical language and information system. 1. Introduction to methodology and encoding rules. Journal of chemical information and computer sciences 1988;28(1):31–36.
26. Szegedy C, Ioffe S, Vanhoucke V, Alemi A. Inception-v4, inception-resnet and the impact of residual connections on learning. In: Proceedings of the AAAI conference on artificial intelligence, vol. 31; 2017. .
27. Touvron H, Bojanowski P, Caron M, Cord M, El-Nouby A, Grave E, et al. Resmlp: Feedforward networks for image classification with data-efficient training. IEEE transactions on pattern analysis and machine intelligence 2022;45(4):5314–5321.
28. Wu Z, Ramsundar B, Feinberg EN, Gomes J, Geniesse C, Pappu AS, et al. MoleculeNet: a benchmark for molecular machine learning. Chemical science 2018;9(2):513–530.

supplementary material

Click here to access/download
Supplementary Material
Supplementary Material.pdf
