# Supplementary material for: Generalizable machine learning models for rapid antimicrobial resistance prediction in unseen health care settings
Source: Gigascience. 2026 Jan 19;15:giaf156. doi: 10.1093/gigascience/giaf156 (PMC12908719; doi:10.1093/gigascience/giaf156)
Supplement: giaf156_Authors_Response_To_Reviewer_Comments_original_submission [file giaf156_authors_response_to_reviewer_comments_original_submission.pdf]

Author's Response To Reviewer Comments

We would like to thank the reviewers for their thorough evaluation of our manuscript and their insightful comments and suggestions. We have addressed all the points raised, and our detailed responses are provided in the attached pdf 'Detailed responses to reviewers'. Corresponding revisions in the main manuscript are highlighted in blue. Additionally, we have expanded the supplementary materials based on the reviewers' feedback.

Cookie Preference Center

We use cookies which are necessary to make our site work. We may also use additional cookies to analyze, improve and personalize our content and your digital experience. For more information, see our Cookie Policy.

You may choose not to allow some types of cookies. However, blocking some types may impact your experience of our site and the services we are able to offer. See the different category headings below to find out more or change your settings.

Allow all

Manage Consent Preferences

Strictly Necessary Cookies

Always active

These cookies are necessary for the website to function and cannot be switched off in our systems. They are usually only set in response to actions made by you which amount to a request for services, such as setting your privacy preferences, logging in or filling in forms. You can set your browser to block or alert you about these cookies, but some parts of the site will not then work.

Functional Cookies

These cookies enable the website to provide enhanced functionality and personalisation. They may be set by us or by third party providers whose services we have added to our pages. If you do not allow these cookies then some or all of these services may not function properly.

Performance Cookies

These cookies allow us to count visits and traffic sources so we can measure and improve the performance of our site. They help us to know which pages are the most and least popular and see how visitors move around the site.

Targeting Cookies

These cookies may be set through our site by our advertising partners. They may be used by those companies to build a profile of your interests and show you relevant adverts on other sites. If you do not allow these cookies, you will experience less targeted advertising.

Cookie List

Clear

Apply Cancel
Consent Leg.Interest

Confirm my choices

Powered by onetrus
