# Supplementary material for: Generalizable machine learning models for rapid antimicrobial resistance prediction in unseen health care settings
Source: Gigascience. 2026 Jan 19;15:giaf156. doi: 10.1093/gigascience/giaf156 (PMC12908719; doi:10.1093/gigascience/giaf156)
Supplement: giaf156_Reviewer_2_Report_original_submission — Johan Lassen, Ph.D. -- 8/18/2025 [file giaf156_reviewer_2_report_original_submission.pdf]

Reviewer Report

Title: Generalizable machine learning models for rapid antimicrobial resistance prediction in unseen healthcare settings

Version: Original Submission **Date:** 8/18/2025

Reviewer name: Johan Lassen, Ph.D.

Reviewer Comments to Author:

This manuscript aims to improve the generalizability of antimicrobial resistance (AMR) prediction models using representation learning. The authors combine masked autoencoders (MAEs) for MALDI-TOF spectra with various antimicrobial encodings, and demonstrate improved performance across hospitals and time periods. While the work is technically sound and addresses an important challenge, I have several questions and suggestions that I believe could strengthen the manuscript.1. Binned Spectra and MAE ArchitectureThe authors use binned MALDI-TOF spectra (6000-dimensional vectors) as input to the MAE. However, it is unclear why raw spectra were not used directly, especially given that contextual patterns in spectral data may be better preserved without binning. Several other studies have successfully made "end-to-end modeling" on raw spectral data (spanning LC-MS, MALDI, and spectroscopy) Could the authors clarify this choice and/or discuss the upsides/downsides?Additionally, the MAE architecture is a simple feedforward network with a single hidden layer. Given the structured nature of spectral data, more expressive architectures (e.g., 1D CNNs or transformers) might better capture local and contextual features. Could the authors justify the use of this architecture over alternatives?2. MAE Training Procedure and Baseline ComparisonThe MAE is trained in an unsupervised fashion to reconstruct masked spectra, and its encoder is later used to generate embeddings for the supervised classifier. This two-stage setup is reasonable, but it raises concern about the baseline comparison.The baseline model uses raw binned spectra without any autoencoding (from what I can see). To isolate the effect of masking, it would be informative to include a comparison to a non-masked autoencoder trained on the same spectra. This would clarify whether the observed improvements stem from representation learning in general or specifically from the masking strategy (this is the question you aim to answer in the abstract/intro).3. Clinical Relevance and Model Use CaseThe manuscript would benefit from a clearer description of how the model would be used in clinical practice. For example, would a clinician input a pathogen spectrum and iterate through candidate drugs to find one with low predicted resistance? Could the model be used to rank antimicrobials or support de-escalation strategies?Clarifying the intended workflow would help contextualize the model's utility and limitations.4. Interpretability and Feature AttributionThe use of a two-stage model (MAE + classifier) limits interpretability. It becomes difficult to trace predictions back to specific spectral features, which is important for understanding biological mechanisms and building clinical trust. Could the authors comment on how interpretability might be recovered in this setup?5. Embedding Size and Representation QualityThe choice of a 512-dimensional embedding for pathogen spectra is not well justified. Could the authors provide a rationale for this size, or include a sensitivity analysis showing how embedding dimensionality affects performance and generalizability?6. Qualitative Analysis of

EmbeddingsThe manuscript could benefit from visualizations indicating the degree of technical bias and whether this is resolved/reduced using the MAE. For example, visualizing the MAE embeddings using UMAP or t-SNE could reveal whether the model captures biological structure (e.g., species or resistance profiles) rather than technical artifacts (e.g., hospital origin). This would strengthen the claim that the representation learning reduces bias and improves generalizability.

Level of Interest

Please indicate how interesting you found the manuscript: Choose an item.

Quality of Written English

Please indicate the quality of language in the manuscript: Choose an item.

Declaration of Competing Interests

Please complete a declaration of competing interests, considering the following questions:

- Have you in the past five years received reimbursements, fees, funding, or salary from an organisation that may in any way gain or lose financially from the publication of this manuscript, either now or in the future?
- Do you hold any stocks or shares in an organisation that may in any way gain or lose financially from the publication of this manuscript, either now or in the future?
- Do you hold or are you currently applying for any patents relating to the content of the manuscript?
- Have you received reimbursements, fees, funding, or salary from an organization that holds or has applied for patents relating to the content of the manuscript?
- Do you have any other financial competing interests?
- Do you have any non-financial competing interests in relation to this paper?

If you can answer no to all of the above, write 'I declare that I have no competing interests' below. If your reply is yes to any, please give details below.

'I declare that I have no competing interests

I agree to the open peer review policy of the journal. I understand that my name will be included on my report to the authors and, if the manuscript is accepted for publication, my named report including any attachments I upload will be posted on the website along with the authors' responses. I agree for my report to be made available under an Open Access Creative Commons CC-BY license (http://creativecommons.org/licenses/by/4.0/). I understand that any comments which I do not wish to be included in my named report can be included as confidential comments to the editors, which will not be published.

Choose an item.

To further support our reviewers, we have joined with Publons, where you can gain additional credit to further highlight your hard work (see: https://publons.com/journal/530/gigascience). On publication of this paper, your review will be automatically added to Publons, you can then choose whether or not to claim your Publons credit. I understand this statement.

Yes Choose an item.
